# Supplementary material for: Fall incidents in nursing home residents: development of a predictive clinical rule (FINDER)
Source: BMJ Open. 2021 May 3;11(5):e042941. doi: 10.1136/bmjopen-2020-042941 (PMC8098923; doi:10.1136/bmjopen-2020-042941)
Supplement: Supplementary data [file bmjopen-2020-042941supp001.pdf]

Supplementary Table 1a: Potential fall risk factors, Model I (day 0)

Risk factors	Mean (SD)	Frequency (number)	% of total	OR	95% C.I. OR		p-value
					Upper	Lower	
**Age (y)**	82.43 (8.46)	-	-	1.044	1.026	1.063	**.000 ***
**Age(y) centred**	0.03 (8.46)	-	-	1.035	1.015	1.055	**.001 ***
**Age(y) centred^2**	71.57 (147.93)	-	-	.998	.996	.999	**.008 ***
**Gender**	-	252 *M*, 572 *F*	30.58 *M*, 69.42 *F*	.561	.415	.758	**.000 ***
**ATC unique**	8.49 (3.91)	-	-	1.035	.999	1.072	.059
**ATC unique≥5**		697	84.59	1.183	.810	1.729	.385
**ATC unique 0-5**		127	15.41	.845	.578	1.235	.385
**ATC unique 5- 10**		399	48.42	.864	.657	1.136	.296
**ATC unique 10-15**		226	27.43	1.103	.811	1.499	.531
**ATC unique ≥15**		72	8.74	1.640	1.000	2.690	**.050 ***
**N06D**		97	11.77	6.057	3.474	10.558	**.000 ***
**C01**		160	19.42	1.168	.827	1.651	.379
**N04A**		1	.12	*NA*	*NA*	*NA*	*NA*
**N06A**		253	30.70	1.394	1.035	1.878	**.029 ***
**N03A**		75	9.10	.499	.304	.820	**.006 ***
**R06A**		32	3.88	1.139	.561	2.313	.719
**N05A**		304	36.89	2.144	1.605	2.863	**.000 ***
**C07**		199	24.15	.864	.628	1.190	.370
**S01ED**		24	2.91	1.414	0.621	3.222	0.409
**N05C**		280	33.98	1.417	1.060	1.894	**.019 ***
**G04**		104	12.62	1.635	1.074	2.489	**.022 ***
**C08**		80	9.71	.895	.564	1.420	.638
**C03**		281	34.10	1.177	.881	1.571	.270
**M03**		7	.85	.000	.000	*NA*	0.999
**C04AC**		0	.00	*NA*	*NA*	*NA*	*NA*
**C01C**		2	.24	1.000	.062	16.055	1.000
**N02A**		101	12.26	.589	.385	.903	**.015 ***
**A02B**		318	38.59	.866	.654	1.147	.316
**C09**		211	25.61	1.039	.760	1.421	.811
**N07C**		20	2.43	1.000	.411	2.435	1.000
**N02B**		395	47.94	1.030	.783	1.355	.834
**N07AA**		3	.36	.498	.045	5.524	.571
**A10**		146	17.72	1.106	.772	1.582	.583
**C02**		6	.73	1.000	.200	4.988	1.000
**M01A**		54	6.55	1.083	.623	1.882	.778
**N07C**		20	2.43	1.000	.411	2.435	1.000
**N05B**		231	28.03	1.352	.996	1.835	**.053 ***
**C10**		176	21.36	1.060	.759	1.479	.734
**R01BA**		0	.00	*NA*	*NA*	*NA*	*NA*
**C01A**		69	8.37	1.100	.671	1.804	.705
**C01B**		8	.97	1.000	.248	4.030	1.000
**C01D**		98	11.89	1.149	.753	1.754	.519
**N04**		37	4.49	.945	.488	1.828	.866
**N06DA**		41	4.98	5.217	2.284	11.917	**.000 ***
**G04CA**		75	9.10	1.768	1.083	2.885	**.023 ***

* statistically significant ($p < 0.05$)

NA = not available; SD = standard deviation; OR = Odds Ratio; C.I.= confidence interval; *M* = male, *F* = female

Supplementary Table 1b: Potential fall risk factors, Model II (day -3)

Risk factors	Mean (SD)	Frequency (number)	% of total	OR	95% C.I. OR		p-value
					Upper	Lower	
**Age (y)**	82.47 (8.39)	-	-	1.043	1.025	1.062	**.000 ***
**Age(y) centred**	0.00 (8.39)	-	-	1.033	1.014	1.054	**.001 ***
**Age(y) centred^2**	70.35 (143.45)	-	-	.998	.996	.999	**.007 ***
**Gender**	-	246 *M*, 570 *F*	30.15% *M*, 69.85% *F*	.546	.403	.740	**.000 ***
**ATC unique**	8.46 (3.90)	-	-	1.029	.993	1.066	.115
**ATC unique≥5**		690	84.56	1.044	.714	1.527	.824
**ATC unique 0-5**		126	15.44	.958	.655	1.401	.824
**ATC unique 5- 10**		401	49.14	.872	.662	1.147	.328
**ATC unique 10-15**		221	27.08	1.057	.776	1.439	.726
**ATC unique ≥15**		68	8.33	1.467	.887	2.428	.136
**N06D**		97	11.89	6.553	3.707	11.584	**.000 ***
**C01**		161	19.73	1.216	.861	1.718	.268
**N04A**		1	.12	*NA*	*NA*	*NA*	*NA*
**N06A**		253	31.00	1.386	1.028	1.868	**.032 ***
**N03A**		75	9.19	.529	.323	.866	**.011 ***
**R06A**		32	3.92	1.133	.558	2.301	.729
**N05A**		304	37.25	2.044	1.530	2.730	**.000 ***
**C07**		199	24.39	.858	.623	1.182	.350
**S01ED**		24	2.94	1.408	.618	3.208	.416
**N05C**		277	33.95	1.365	1.020	1.828	**.036 ***
**G04**		104	12.75	1.554	1.022	2.362	**.039 ***
**C08**		81	9.93	.916	.579	1.450	.708
**C03**		278	34.07	1.132	.847	1.512	.404
**M03**		7	.86	.000	.000	*NA*	.999
**C04AC**		0	.00	*NA*	*NA*	*NA*	*NA*
**C01C**		2	.25	.994	.062	15.954	.996
**N02A**		95	11.64	.568	.366	.883	**.012 ***
**A02B**		314	38.48	.840	.634	1.115	.228
**C09**		211	25.86	1.059	.774	1.449	.720
**N07C**		19	2.33	1.107	.444	2.764	.827
**N02B**		384	47.06	.933	.709	1.229	.624
**N07AA**		3	.37	.495	.045	5.489	.567
**A10**		143	17.52	1.119	.779	1.608	.541
**C02**		6	.74	.994	.199	4.958	.994
**M01A**		53	6.50	.955	.547	1.667	.871
**N07C**		19	2.33	1.107	.444	2.764	.827
**N05B**		229	28.06	1.380	1.015	1.877	**.040 ***
**C10**		179	21.94	1.037	.745	1.446	.828
**R01BA**		0	.00	*NA*	*NA*	*NA*	*NA*
**C01A**		68	8.33	1.133	.688	1.865	.625
**C01B**		8	.98	.994	.247	4.006	.993
**C01D**		100	12.25	1.194	.784	1.816	.409
**N04**		37	4.53	.940	.486	1.818	.853
**N06DA**		40	4.90	6.071	2.519	14.628	**.000 ***
**G04CA**		75	9.19	1.653	1.015	2.689	**.043 ***

* statistically significant (p-value < 0.05)

NA = not available; SD = standard deviation; OR = Odds Ratio; C.I.= confidence interval; *M* = male, *F* = female

Supplementary Table 1c: Potential fall risk factors, Model III (day -5)

Risk factors	Mean (SD)	Frequency (number)	% of total	OR	95% C.I. OR		p-value
					Upper	Lower	
**Age (y)**	82.52 (8.36)	-	-	1.042	1.024	1.061	**.000 ***
**Age(y) centred**	0.00 (8.36)	-	-	1.033	1.013	1.053	**.001 ***
**Age(y) centred^2**	69.75 (143.05)	-	-	.998	.996	.999	**.010 ***
**Gender**	-	243 *M*, 569 *F*	29.93 *M*, 70.07 *F*	.536	.395	.728	**.000 ***
**ATC unique**	8.44 (3.93)	-	-	1.023	.987	1.059	.214
**ATC unique≥5**		684	84.24	1.083	.742	1.581	.679
**ATC unique 0-5**		128	15.76	.923	.633	1.348	.679
**ATC unique 5- 10**		395	48.65	.906	.688	1.194	.484
**ATC unique 10-15**		223	27.46	1.083	.796	1.474	.613
**ATC unique ≥15**		66	8.13	1.296	.781	2.152	.316
**N06D**		94	11.58	6.833	3.801	12.285	**.000 ***
**C01**		160	19.70	1.198	.847	1.695	.306
**N04A**		1	.12	*NA*	*NA*	*NA*	*NA*
**N06A**		251	30.91	1.357	1.006	1.830	**.045 ***
**N03A**		74	9.11	.541	.330	.888	**.015 ***
**R06A**		30	3.69	.995	.480	2.063	.989
**N05A**		298	36.70	1.973	1.475	2.639	**.000 ***
**C07**		196	24.14	.868	.629	1.198	.391
**S01ED**		24	2.96	1.408	.618	3.208	.416
**N05C**		273	33.62	1.400	1.045	1.878	**.024 ***
**G04**		103	12.68	1.457	.958	2.215	.078
**C08**		78	9.61	.940	.589	1.499	.794
**C03**		277	34.11	1.120	.837	1.498	.446
**M03**		7	.86	.000	.000	*NA*	.999
**C04AC**		0	.00	*NA*	*NA*	*NA*	*NA*
**C01C**		2	.25	.994	.062	15.955	.996
**N02A**		91	11.21	.586	.374	.916	**.019 ***
**A02B**		312	38.42	.840	.633	1.114	.226
**C09**		209	25.74	1.060	.773	1.451	.719
**N07C**		19	2.34	1.107	.444	2.764	.827
**N02B**		383	47.17	.905	.687	1.194	.481
**N07AA**		3	.37	.495	.045	5.489	.567
**A10**		140	17.24	1.065	.739	1.534	.736
**C02**		6	.74	.994	.199	4.959	.994
**M01A**		54	6.65	.918	.528	1.597	.761
**N07C**		19	2.34	1.107	.444	2.764	.827
**N05B**		228	28.08	1.332	.980	1.812	.067
**C10**		175	21.55	1.038	.743	1.451	.826
**R01BA**		0	.00	*NA*	*NA*	*NA*	*NA*
**C01A**		68	8.37	1.133	.688	1.865	.625
**C01B**		8	.99	.994	.247	4.006	.993
**C01D**		99	12.19	1.168	.766	1.781	.469
**N04**		37	4.56	.940	.486	1.818	.853
**N06DA**		39	4.80	7.307	2.827	18.885	**.000 ***
**G04CA**		75	9.24	1.555	.958	2.524	.074

* statistically significant (p-value < 0.05)

NA = not available; SD = standard deviation; OR = Odds Ratio; C.I.= confidence interval; *M* = male, *F* = female

Milosevic V, *et al. BMJ Open* 2021; 11:e042941. doi: 10.1136/bmjopen-2020-042941
