# Supplementary material for: Fall incidents in nursing home residents: development of a predictive clinical rule (FINDER)
Source: BMJ Open. 2021 May 3;11(5):e042941. doi: 10.1136/bmjopen-2020-042941 (PMC8098923; doi:10.1136/bmjopen-2020-042941)
Supplement: Author's manuscript [file bmjopen-2020-042941.draft_revisions.pdf]

BMJ Open is committed to open peer review. As part of this commitment we make the peer review history of every article we publish publicly available.

When an article is published we post the peer reviewers' comments and the authors' responses online. We also post the versions of the paper that were used during peer review. These are the versions that the peer review comments apply to.

The versions of the paper that follow are the versions that were submitted during the peer review process. They are not the versions of record or the final published versions. They should not be cited or distributed as the published version of this manuscript.

BMJ Open is an open access journal and the full, final, typeset and author-corrected version of record of the manuscript is available on our site with no access controls, subscription charges or pay-per-view fees (http://bmjopen.bmj.com).

If you have any questions on BMJ Open's open peer review process please email info.bmjopen@bmj.com

Fall Incidents in Nursing home patients: DEvelopment of a predictive clinical Rule (FINDER)

Journal:	*BMJ Open*
Manuscript ID	bmjopen-2020-042941
Article Type:	Original research
Date Submitted by the Author:	22-Jul-2020
Complete List of Authors:	Milosevic, Vanja; Elkerliek Ziekenhuis, Clinical Pharmacy; Zuyderland Medical Centre Sittard-Geleen, Clinical Pharmacy, Pharmacology and Toxicology<br>Linkens, Aimee; Maastricht University Medical Centre+, Internal Medicine<br>Hurkens, Kim; Zuyderland Medisch Centrum Heerlen, Geriatric Medicine, Department of Internal Medicine<br>Wong, Dennis; Zuyderland Medical Centre Sittard-Geleen, Clinical Pharmacy, Pharmacology and Toxicology<br>Winkens, Bjorn; Maastricht University, Methodology and Statistics<br>van Oijen, Brigit; Zuyderland, Clinical Pharmacy, Pharmacology and Toxicology<br>Mestres-Gonzalvo, Carlota; Maastricht University Medical Centre+, Clinical Pharmacy and Toxicology<br>van der Kuy, Hugo; Erasmus MC, Clinical Pharmacy
Keywords:	CLINICAL PHARMACOLOGY, GENERAL MEDICINE (see Internal Medicine), GERIATRIC MEDICINE

I, the Submitting Author has the right to grant and does grant on behalf of all authors of the Work (as defined in the below author licence), an exclusive licence and/or a non-exclusive licence for contributions from authors who are: i) UK Crown employees; ii) where BMJ has agreed a CC-BY licence shall apply, and/or iii) in accordance with the terms applicable for US Federal Government officers or employees acting as part of their official duties; on a worldwide, perpetual, irrevocable, royalty-free basis to BMJ Publishing Group Ltd ("BMJ") its licensees and where the relevant Journal is co-owned by BMJ to the co-owners of the Journal, to publish the Work in this journal and any other BMJ products and to exploit all rights, as set out in our licence.

The Submitting Author accepts and understands that any supply made under these terms is made by BMJ to the Submitting Author unless you are acting as an employee on behalf of your employer or a postgraduate student of an affiliated institution which is paying any applicable article publishing charge ("APC") for Open Access articles. Where the Submitting Author wishes to make the Work available on an Open Access basis (and intends to pay the relevant APC), the terms of reuse of such Open Access shall be governed by a Creative Commons licence – details of these licences and which Creative Commons licence will apply to this Work are set out in our licence referred to above.

Other than as permitted in any relevant BMJ Author's Self Archiving Policies, I confirm this Work has not been accepted for publication elsewhere, is not being considered for publication elsewhere and does not duplicate material already published. I confirm all authors consent to publication of this Work and authorise the granting of this licence.

For peer review only

Title

Fall Incidents in Nursing home patients: DEvelopment of a predictive clinical Rule (FINDER)

Authors

Vanja Milosevic[1,2]
vmilosevic@elkerliek.nl
Aimee Linkens[3]
aimee.linkens@mumc.nl
Kim Hurkens[4]
k.hurkens@zuyderland.nl
Dennis Wong[1]
d.wong@zuyderland.nl
Bjorn Winkens[5]
bjorn.winkens@maastrichtuniversity.nl
Brigit van Oijen[1]
br.vanoijen@zuyderland.nl
Carlota Mestres-Gonzalvo[6]
c.mestres@mumc.nl
Hugo van der Kuy[7]
h.vanderkuy@erasmusmc.nl

[1]Department of Clinical Pharmacy, Pharmacology and Toxicology, Zuyderland Medical Centre, Dr. H van der Hoffplein 1, 6162 BG, Sittard-Geleen and Henri Dunantstraat 5, 6419 PC Heerlen, The Netherlands.
[2]Department of Clinical Pharmacy, Elkerliek Hospital, Wesselmanlaan 25, 5707 HA Helmond, The Netherlands.
[3]Department of Internal Medicine, Maastricht University Medical Centre+, P. Debyelaan 25, 6229 HX Maastricht, The Netherlands.
[4]Department of Internal Medicine sect. of Geriatric Medicine, Zuyderland Medical Centre, Dr. H van der Hoffplein 1, 6162 BG, Sittard-Geleen and Henri Dunantstraat 5, 6419 PC Heerlen, The Netherlands.
[5]Department of Methodology and Statistics, Faculty of Health, Medicine and Life Sciences, Maastricht University, P. Debyeplein 1, 6229 HA Maastricht, The Netherlands.

For peer review only

For peer review only - http://bmjopen.bmj.com/site/about/guidelines.xhtml

[6]Department of Clinical Pharmacy and Toxicology, Maastricht University Medical Centre+, P. Debyelaan 25, 6229 HX Maastricht, The Netherlands.

[7]Department of Clinical Pharmacy, Erasmus Medical Centre, Postbus 2040, 3000 CA Rotterdam, The Netherlands.

Corresponding Author:

Prof. dr. Hugo van der Kuy

Department of Clinical Pharmacy

Erasmus Medical Centre

Postbus 2040

3000 CA Rotterdam

The Netherlands

Email: h.vanderkuy@erasmusmc.nl

Keywords

Fall risk, Clinical Rule, nursing home patients.

Word count: 3.567 (without abstract, strength and limitations, literature, figures and tables)

Number of tables: 4

Number of figures: 2

Number of supplementary tables: 3 (1a,b and c)

Number of supplementary texts: 0

For peer review only

For peer review only - http://bmjopen.bmj.com/site/about/guidelines.xhtml

ABSTRACT (294)

Objectives: To develop (part I) and validate (part II) an electronic fall risk clinical rule (CR) to identify nursing home patients (NH-patients) at risk for a fall incident.

Design: Observational, retrospective database study.

Setting: Nursing homes

Participants: A total of 1668 (824 in part I, 844 in part II) NH-patients from The Netherlands were included. Data of participants from part I were excluded in part II.

Primary and secondary outcome measures: development and validation of a fall risk CR in NH-patients. Logistic regression analysis was conducted to identify the fall risk-variables in part I. With these, three CRs were developed (i.e. at the day of the fall incident and three days and five days prior to the fall incident). The overall prediction quality of the CRs were assessed using the Area Under the Receiver Operating Characteristics (AUROC), and a cut-off value was determined for the predicted risk ensuring a sensitivity ≥ 0.85. Finally, one CR was chosen and validated in part II using a new retrospective dataset.

Results: Eleven fall risk-variables were identified in part I. The AUROCs of the three CRs form part I were similar: the AUROC for model I, II and III were 0.714 (95% CI 0.679 to 0.748), 0.715 (95%CI 0.680 to 0.750) and 0.709 (95%CI 0.674 to 0.744), respectively. Model III (i.e. 5 days prior to the fall incident) was chosen for validation in part II. The validated AUROC of the CR, obtained in part II, was 0.603 (95%CI 0.565 to 0.641) with a sensitivity of 83.41% (95% CI 79.44% to 86.76%) and a specificity of 27.25%(95% CI 23.11% to 31.81%).

Conclusion: Medication data and patient characteristics only are not sufficient enough to develop a successful CR with a high sensitivity and specificity to predict fall risk in NH-patients.

Trial registration: not available

STRENGHTS AND LIMITATIONS OF THIS STUDY

- Currently there is no golden standard in the Netherlands regarding the use of a specific tool in the prevention of fall risk in nursing home patients.
- This study was based on electronic data and variables that are currently available in daily practice and therefore easy to implement. However, fall risk factors that were not available electronically are lacking.

For peer review only - http://bmjopen.bmj.com/site/about/guidelines.xhtml

- The clinical rule was developed and validated using two different retrospective data sets of the same nursing homes and therefore lacks a prospective validation in a different nursing home setting.

FUNDING

This research received no specific grant from any funding agency in the public, commercial or not-for-profit sectors.

Competing Interests

All authors have declared that no competing interests exist.

Abbreviations

Clinical Decision Support System (CDSS), fall risk increasing drugs (FRIDs), receiver operating characteristics (ROC), area under the receiver operating characteristics (AUROC), Positive Predictive Value (PPV), Negative Predictive Value (NPV)

For peer review only

For peer review only - http://bmjopen.bmj.com/site/about/guidelines.xhtml

INTRODUCTION / BACKGROUND (707)

Falls are common in elderly people aged 65 years and over. According to the World Health Association, 28-35% of the elderly fall each year [1]. This percentage increases with age (e.g. 32-42% in the age group of 70 years and older) as does the fatal falls rate [2-4]. However, not only age seems to be related to the risk of falls, the frailty level is also a risk factor. For example, elderly who live in nursing homes have higher falling rates compared to those living in the community [5]. The incidence of falls in nursing home patients ranges from 30 to 50% per year [1,6].

Worldwide there are 646,000 falls every year which result in death, making falls the second leading cause of unintended death [1]. This fact along with the ageing population challenges us with the question: how to prevent falls?

There are four different dimensions involved in fall incidents: biological (e.g. race, gender, age, (multi)-morbidity), environmental (e.g. building design, insufficient light), socio-economic (e.g. inadequate housing, limited access to social services and health), and behavioural factors (e.g. polypharmacy, inappropriate foot ware) [1].

Focusing on behavioural aspects, specifically on medication, there are several studies conducted to investigate the effect of medication on fall risk [1,7-20]. These studies have shown that the risk of falls resulting in injuries and/or death increases with increasing number of used drugs in elderly [7]. Other studies investigated the association of different drug classes with an increased risk of falls [11-20]. Examples of identified drug classes with an increased fall risk are anti-arrhythmic drugs, antidepressants, psychotropic drugs, and diuretics [1,8-11].

Different strategies and tools to prevent fall incidents are developed or still in development (e.g. STRATIFY, Hendrich Fall Risk Model) [21]. However, the multifactorial and complex variables of fall incidents make it extremely difficult to develop a tool with a high sensitivity and specificity that is easy to implement in practice [1,21,22].

The current tools differ in number and type of fall risk factors included in the tool (e.g. multifactorial assessment tools, functional mobility or medication related assessment tool). Also, the applicability is often restricted to one specific setting and or subpopulation (e.g. hospital, nursing home, ambulatory). In The Netherlands, there is still no recommendation for

For peer review only

For peer review only - http://bmjopen.bmj.com/site/about/guidelines.xhtml

one specific tool in the prevention of fall risk in nursing home patients, leading to local differences regarding fall risk management.

Of the most common tools for the prediction of fall risk (i.e. Hendrich Fall Risk Mode I (and II)(HFRM I and II), St. Thomas's risk assessment tool in falling elderly inpatients (STRATIFY) and the Morse Fall Scale (MFS), only the HFRM II tool makes use of information regarding anti-epileptics and benzodiazepines. Both MFS and STRATIFY have no medication related fall risk factors included in the tool [6,23].

Nunan et al. [22], conducted a systematic review regarding fall risk assessment tools in the long-term care setting. In general, the following remarks can be made with the current tools in the prevention of fall incidents: they are either not validated, or have a low sensitivity and/or specificity (lower than 70%), and/or the practical applicability is below the desired level [22]. Therefore, there is still room for improvement.

Given the multifactorial aspect of fall incidents, a tool that combines all of the above described dimensions involved in fall incidents, would probably give the best results. Currently there is no such tool available.

Our goal is to focus on all of the electronically available data of interest in fall incidents, aiming to combine as many different dimensions involved in fall incidents as possible. Thereby we will include age and gender, the presence of specific ATC-codes, the number of unique ATC-codes and laboratory parameters. The specific ATC-codes and laboratory parameters that will be included in this study, are those from which there is evidence from other studies that there is a (possible) association with the fall risk.

With a Clinical Decision Support System (CDSS), one is able to combine different data, such as patient characteristics, laboratory and medication data, with the goal to generate specific alerts per patient. Developing and implementing a predictive clinical rule for fall risk in an electronic CDSS could therefore be helpful for healthcare professionals in order to prevent falls in those who are at increased risk.

Objective (38)

The objective of this study is to develop (part I) and validate (part II) a fall risk clinical rule that can be used in an electronic CDSS to identify nursing home patients at risk for a fall incident.

For peer review only

For peer review only - http://bmjopen.bmj.com/site/about/guidelines.xhtml

METHODS (1029)

Design (69)

An observational retrospective database study was conducted in two parts. In part I, a predictive fall risk clinical rule for implementation in an electronic CDSS was developed in a cohort of nursing home patients. In part II, the developed clinical rule was retrospectively validated in a different cohort of nursing home patients.

For both parts of the study, data from nursing home patients from Sittard-Geleen, The Netherlands, were used.

Patients selection (38)

All patients living in NH were selected. The population consists of both somatic and non-somatic patients. No exclusion criteria were used. Only patients that were identified as faller in the first part were excluded in the second part.

Outcome (131)

The outcome that is predicted by the fall risk clinical rule is a fall.

In part I, nursing home patients who have fallen at least once within the period of January 25th 2011 until December 31th 2011, were defined as *fallers*. Nursing home patients who have not fallen in the same period, were defined as *non-fallers*. For part II the same design was used as in part I. Part II was conducted in a new cohort of nursing home patients in the period of January 25th 2016 until December 31th 2016.

For both parts of the study, the date of the first fall of the faller within the study period was set as reference date. On this specific reference date, a control patient was selected at random from the non-fallers group.

Potential predictors (239)

Hospital and nursing home electronic systems were used to extract data on patient characteristics (i.e. age and gender), laboratory data, medication (number and type of

For peer review only

For peer review only - http://bmjopen.bmj.com/site/about/guidelines.xhtml

medicines), and reports on fall incidents. More specifically, data on medication were collected at the reference date and 3 and 5 days prior to the reference date, while laboratory data of approximately up to 3 months prior to the reference date were retrieved.

The laboratory parameters intended for analysis are known from literature to be associated with fall risk: albumin, sodium, potassium, creatinine, kidney clearance (MDRD), glucose and hemoglobin [24-29]. Regarding the medication data, the patients medication list was screened for presence of 35 different ATC-codes all of which are shown to have a (possible) association with fall risk [6,8-20]. Also the number of unique ATC-codes was collected.

Finally, electronically available patient characteristics, such as age and gender at time of the reference date, as well as the reports on fall incidents (date of the report) were obtained.

The above described dataset for part I was analysed in order to develop a predictive fall risk clinical rule for an electronic CDSS based on relevant risk factors for the prediction of the fall risk in nursing home patients. In part II the developed clinical rule was validated by determining its specificity and sensitivity using a new dataset of Zuyderland MC nursing home patients obtained in the period of January 25th 2016 until December 31th 2016.

Statistical analysis (271)

Statistical analyses were performed using IBM SPSS Statistics for Windows (version 21.0. Armonk, NY: IBM Corp.). Two-sided p-values smaller than or equal to 0.05 were considered statistically significant.

For part I of the study, logistic regression analysis was used to identify statistically significant risk factors for the prediction of the fall risk, where variables significantly related to the outcome were included in the multiple logistic regression model. Linearity assumption was checked for numerical variables by centering these variables and adding the quadratic centred term to the model. If this quadratic term was significant, the linear as well as quadratic term for this variable were included in the multiple regression model. The predictive clinical rule (multiple linear regression model) was then used to obtain a predictive score which quantifies the estimated fall risk of the individual nursing home patient according to his/her specific risk factors. The bootstrap method was used (1000 bootstrap datasets) to obtain a shrinkage factor in order to correct for over-fitting. The overall predictive quality of the clinical rule was

For peer review only

For peer review only - http://bmjopen.bmj.com/site/about/guidelines.xhtml

determined by the c-statistic, i.e. the area under the receiver operating characteristics (AUROC) curve. Additionally, an optimal cut-off point for the estimated fall risk was obtained by two criteria: A) maximizing the Jouden's J-statistics, i.e. sensitivity+specificity-1, and B) maximizing specificity where the sensitivity is at least 85%.

The predictive clinical rule was applied to the dataset from part II of the study, where the optimal cut-off value obtained from part I was then used to determine the validated prognostic values of the clinical rule. To obtain 95% confidence intervals around these prognostic values an online calculator (http://vassarstats.net/clin1.html) was used.

Sample size calculation (240)

The sample size was estimated for a clinical rule that could consist of 30-40 risk factors. This number of risk factors was based on the number of different ATC-codes (fall risk increasing drugs (FRIDs), known from literature), use of polypharmacy (≥5 different ATC-codes in chronic use), the number of relevant laboratory data, and the patient characteristics age and gender [6,8-20,24-29]. The multiple logistic regression model requires at least 10 fallers for each individual risk factor. In order to obtain a representative incidence of fallers (about 50% per year) in the study population, also 10 non-fallers are needed per individual risk factor. This means that a total of approximately 800 nursing home patients (at least 400 fallers and 400 non-fallers) were needed to carry out part I of the study, and another 800 nursing home patients were needed for part II of the study (at least 400 fallers and 400 non-fallers).

These numbers would be large enough to ensure precise estimates of sensitivity and specificity of the predictive clinical rule. More specifically, the width of the 95% confidence interval (CI) of sensitivity and specificity of the predictive clinical rule would be maximally equal to $2*1.96*0.5/\sqrt{n} = 1.96/\sqrt{n}$, where n equals the number of fallers for sensitivity and number of non-fallers for specificity. Therefore, with at least 400 fallers and 400 non-fallers, the 95%CI of sensitivity and specificity of the predictive clinical rule would not be wider than 0.10 ($1.96/\sqrt{400} = 0.098$).

For peer review only

For peer review only - http://bmjopen.bmj.com/site/about/guidelines.xhtml

Patient and Public Involvement (20)

Patients or the public were not involved in the design, or conduct, or reporting, or dissemination plans of our research.

RESULTS (446)

Participants

A total of 824 nursing home patients (412 fallers and 412 non-fallers) in the time period of January 25th 2011 up to December 31th 2011 were included in part I of the study (Figure 1). Characteristics of the included nursing home patients for part I of the study are shown in Table 1.

Table 1: Patient characteristics in part I of the study

	Model I (day 0)			Model II (day -3)			Model III (day -5)		
	Faller (n=412)	Non-faller (n=412)	Total (n=824)	Faller (n=409)	Non-faller (n=407)	Total (n=816)	Faller (n=407)	Non-faller (n=405)	Total (n=812)
**Mean Age ± SD* (y)**	83.91 ±6,87	80.96 ±9,59	82.43 ±8,46	83.89 ±6,86	81.05 ±9,49	82.47 ±8,39	83.90 ±6,88	81.13 ±9,42	82.52 ±8,36
**Male**	151 (36.7%)	101 (24.5%)	252 (30.6%)	149 (36.4%)	97 (23.8%)	246 (30.1%)	148 (36.4%)	95 (23.5%)	243 (29.9%)
**Female**	261 (63.3%)	311 (75.5%)	572 (69.4%)	260 (63.6%)	310 (76.2%)	570 (69,9%)	259 (63.6%)	310 (76.5%)	569 (70.1%)

*SD= standard deviation; y =years

Model development and specification

Table 2 shows the variables that were selected from literature and included for analysis in part I of the study. Three predictive models were created as medication data were collected at three different moments, i.e. at time of the reference date (model I), 3 days prior to the reference date (model II), and 5 days prior to the reference date (model III). As laboratory data (collected up to approximately 3 months prior to the reference date) were only available in 411 out of 824 nursing home patients (49.9%), the models were only based on medication data (specific ATC codes), age and gender.

For peer review only - http://bmjopen.bmj.com/site/about/guidelines.xhtml

Table 2: Fall risk variables

Variable	Description
**Polyfarmacy**	
ATCunique	Number of unique ATC codes
ATCunique ≥5	Presence of 5 or more unique ATC codes (yes=1, no=0)
ATCunique 0 - 5	Presence of 0-5 unique ATC codes (yes=1, no=0)
ATCunique 5 - 10	Presence of 5-10 unique ATC codes (yes=1, no=0)
ATCunique10 - 15	Presence of 10-15 unique ATC codes (yes=1, no=0)
ATCunique ≥ 15	Presence of 15 or more unique ATC codes (yes=1, no=0)
**Patient characteristics**	
Gender	Male / Female
Age	Age in years
Age_c	Age in years, centered
Age_c2	Age in years, centered quadratic term
**Presence of specific ATC codes (yes=1, no=0)**	
A02B	DRUGS FOR PEPTIC ULCER AND GASTRO-OESOPHAGEAL REFLUX DISEASE (GORD)
A10	DRUGS USED IN DIABETES
C01	CARDIAC THERAPY
C01A	CARDIAC GLYCOSIDES
C01B	ANTIARRHYTHMICS, CLASS I AND III
C01C	CARDIAC STIMULANTS EXCL. CARDIAC GLYCOSIDES
C01D	VASODILATORS USED IN CARDIAC DISEASES
C02	ANTIHYPERTENSIVES
C03	DIURETICS (cardiovascular system)
C04AC	Nicotinic acid and derivatives (peripheral vasodilators)
C07	BETA BLOCKING AGENTS (cardiovascular system)
**C08**	CALCIUM CHANNEL BLOCKERS (cardiovascular system)
**C09**	AGENTS ACTING ON THE RENIN-ANGIOTENSIN SYSTEM
**C10**	LIPID MODIFYING AGENTS
G04	UROLOGICALS
G04CA	Alpha-adrenoreceptor antagonists (urologicals)
M01A	ANTIINFLAMMATORY AND ANTIRHEUMATIC PRODUCTS, NON-STEROIDS
M03	MUSCLE RELAXANTS
N02A	OPIOIDS (analgetics)
N02B	OTHER ANALGESICS AND ANTIPYRETICS
N03A	ANTIEPILEPTICS
N04	ANTI-PARKINSON DRUGS
N04A	ANTICHOLINERGIC AGENTS (Anti-parkinson drugs)
N05A	ANTIPSYCHOTICS
N05B	ANXIOLYTICS
N05C	HYPNOTICS AND SEDATIVES
N06A	ANTIDEPRESSANTS
N06D	ANTI-DEMENTIA DRUGS
N06DA	Anticholinesterases (psychoanaleptics)
N07AA	Anticholinesterases (parasympathomimetics)
N07C	ANTIVERTIGO PREPARATIONS
R01BA	Sympathomimetics (respiratory system)
R06A	ANTIHISTAMINES FOR SYSTEMIC USE
S01ED	Beta blocking agents (ophthalmologicals)

For peer review only

For peer review only - http://bmjopen.bmj.com/site/about/guidelines.xhtml

From the 44 variables studied in total (Table 2), only 11 variables were found to be significantly related to fall risk (supplementary table 1a,b,c). The multiple logistic regression models consisting of these 11 variables were applied to the data. The AUROC for model I, II and III were 0.714 (95% CI 0.679-0.748), 0.715 (95%CI 0.680-0.750) and 0.709 (95%CI 0.674-0.744), respectively.

For the development of the predictive clinical rule, model III (5 days before reference day) was chosen, since the predictive quality of models I, II and III were similar. Based on the significant fall risk variables, and after correcting for over-fitting (shrinkage factor = 0.8979), the following predictive clinical rule was developed to calculate the fall risk (see Table 3).

Table 3: Fall risk predictive algorithm (*clinical rule*) for model III (5 days prior to reference day).

Fall risk predictive clinical rule (model III) in nursing home patients:
Fall risk = 1/(1+Exp(-Linear predictor).

Linear predictor = 0.7799 + *0.8979**(-0.6802*V1 + 0.0460*V2- -0.0016*V3+ 1.6442*V4 + 0.2664*V5 -0.4763*V6 + 0.4267*V7 + 0.2368*V8 + 0.0001*V9 -0.3780*V10+0.2927*V11)
(shrinkage factor =0.8979)

Predictive fall risk factors:

V1	Gender (1 = male, 2 = female)
V2	Age_c = age -82,52 (age centered, linear term)
V3	Age_c2 = $(\text{age\_c})^2$ (age centered, quadratic term)
V4	Presence of medicines with ATC-code NO6D (yes=1, no=0)
V5	Presence of medicines with ATC-code N06A (yes=1, no=0)
V6	Presence of medicines with ATC code N03A (yes=1, no=0)
V7	Presence of medicines with ATC code N05A (yes=1, no=0)
V8	Presence of medicines with ATC code N05C (yes=1, no=0)
V9	Presence of medicines with ATC code G04 (yes=1, no=0)
V10	Presence of medicines with ATC code N02A (yes=1, no=0)
V11	Presence of medicines with ATC code N05B (yes=1, no=0)

The optimal cut-off value for the fall risk using Jouden's J-statistics was 0.5726, resulting in a sensitivity of 46.9% and a specificity of 81.7%. If the optimal cut-off value was based on the second criterion, where the sensitivity should at least be 85.0%, then the associated cut-off value was 0.4303, resulting in a sensitivity of 85.0% and a specificity of 40.7%.

For peer review only

Model performance

The developed clinical rule was validated in a cohort of 844 nursing home patients (422 fallers and 422 non-fallers) in the time period of January 25th, 2016 to December 31th, 2016. Applying the predictive clinical rule on this cohort of nursing home patients resulted in an AUROC of 0.603 (95%CI 0.565-0.641) (see Figure 2). If the cut-off value for the fall risk was based on the second criterion (sensitivity ≥ 85%), then the validated sensitivity and specificity were 83.4% (95% CI 79.4% - 86.8%) and 27.3% (95% CI 23.1%-31.8%), respectively. The estimates of the diagnostic values with corresponding 95% confidence intervals for this model and cut-off value are shown in Table 4.

Table 4: Estimates of the sensitivity, specificity, PPV and NPV of the validation set

	Estimated value	95% confidence interval	
		Lower limit	Upper limit
**Sensitivity**	83.4%	79.4%	86.8%
**Specificity**	27.3%	23.1%	31.8%
**PPV***	53.4%	49.5%	57.3%
**NPV***	62.2%	54.7%	69.1%

* PPV (Positive Predictive Value); NPV (Negative Predictive Value)

DISCUSSION AND CONCLUSION (1322)

A clinical rule for nursing home patients was developed and validated. This resulted in a clinical rule which can be used to predict the fall risk 5 days prior to the fall incident, based on 11 variables, which were found to be significantly related to fall risk as shown in part I of the study. After identification of the 11 variables, three prediction models were compared: a fall risk prediction model at the reference date, 3 days prior to the reference date and 5 days prior to the reference date. Since the AUROC of all three models was similar, we choose to validate (in part II) the model to predict the fall incidence 5 days prior to the actual event. Because in daily practice it is preferred to predict the fall incident as early on as possible in order to be able to take the necessary action.

For peer review only

Since fall incidents are a massive problem in society and often result in a hospital admission or high mortality rates, we have chosen to set the sensitivity of the prediction model at 85.0%. A sensitivity of 85.0% results in missing less fall incidents.

A critical remark is that validation of the clinical rule (part II of the study) resulted in 83.4% sensitivity and 27.3% specificity. A specificity of 27.3% means that approximately 73% of the patients who would not fall within 5 days would incorrectly be classified as having an increased fall risk. Since nursing home patients are vulnerable, it may be useful to review the medication regardless of the fall risk. Also, the actions that are taken in fall risk prevention are mostly not drug related but include e.g. exercise therapy, psychological health, vision, environmental conditions, diet and nutrition and sometimes may include medication [30]. In addition, it is still up to the healthcare professional to review per individual whether actions are needed to be taken to prevent falls and if so, which actions need to be taken, customized to the subject's needs.

The low specificity after validation of the clinical rule, is probably due to the differences between both datasets (part I and II of the study). The ratio between males and females is different between the two datasets, also the presence of specific ATC codes is different. In addition, the medication dosage and the duration of use is not included in the clinical rule, which may vary between the two datasets.

The rather low AUROC for the prediction model, means that the model is unsuccessful at distinguishing between classes (here: fallers and non-fallers). The optimal AUROC value would be 1.0. In the current study, we found an AUROC of 0.603 (95%CI 0.565-0.641). The low AUROC is probably due to the fact that we choose to base the clinical rule on electronically available data only in order to make it more suitable for implementation in daily practice. Making this choice also means that it is not possible to implement important risk factors, like frailty, in the clinical rule. The low AUROC therefore confirms the complex and multifactorial aspects of the risk of fall incidents, since only a few (i.e. gender, age and medication related data) of the fall risk factors were included in the predictive clinical rule.

Besides the included fall risk factors in the developed predictive clinical rule, the intention was to include laboratory data as well in order to get the most out of the electronically available data. Given the fact that laboratory checks are not performed on daily basis in the nursing home setting, we choose to collect laboratory data up to three months prior to the date of the fall incident. We intended to consider only the most recent known laboratory value of the specific laboratory parameter in the analysis. However, only in 411 out of the 824 nursing home patients laboratory data, for at least one of the laboratory parameters

For peer review only

For peer review only - http://bmjopen.bmj.com/site/about/guidelines.xhtml

intended for analysis, was available. Taking these data into account in the analysis would result in a substantial loss of power. Therefore, no association of laboratory data with fall risk were studied.

Regarding the medication data, the presence of specific ATC-codes in the nursing home patients medication list was checked. This was carried out at three specific moments: at the day of the fall incident, three days prior to the fall incident and five days prior to the fall incident. Since the association between the number of medication, type of medication and the risk of falls was studied, medication in use close at the time of the fall incident, was collected. It is difficult to define what the exact time period should be, because the effect of a specific medicine on a fall is possibly related to the half-life of the drug but could also result from drug-drug interactions. The half-life of the drug and the presence or absence of drug-drug interactions is different for every patient, therefore we chose to compare the association at the time of the fall incident and three and five days prior to the fall incident.

Regarding the results on the association of specific ATC codes with fall risk, we only found NO6D (anti-dementia drugs), N06A (antidepressants), N03A (antiepileptics), N05A (antipsychotics), N05C (hypnotics and sedatives), G04 (urologicals), N02A (opioids) and N05B (anxiolytics) to be significantly associated. The other ATC codes were not significant despite their found association in other studies [6,8-20]. However, this could also be due to the fact that some of the ATC codes were not present at all or only for a small group of nursing home patients. Furthermore, in our study polypharmacy was not statistically significantly related to fall risk. Although there are studies that show that polypharmacy increases the risk of falls, a meta-analysis showed that from the 19 studies that investigated the association of polypharmacy in older people with the risk of falls, only 6 studies reported that polypharmacy increases the risk of falling also the odds ratio reporting this association was nearing 1.0 [31]. The question therefore arises whether polypharmacy itself, or only the number of fall risk increasing drugs is a fall risk increasing variable. Further research is needed to investigate this.

The study has a few limitations. The dataset of the included nursing home patients consisted of fallers and non-fallers. The fallers were classified as faller based on the fall reports. It is unknown whether these fall reports are complete and which definition of the fall incident is used for reporting. It is also known that fall reports which are not fully completed, are not reported. In other words, the number of actual fallers is probably underestimated. With regards to the definition of the fall incidents used for reporting, we believe, that , this does not affect the outcome, since this bias occurs in both groups (fallers and non-fallers).

For peer review only

Another limitation of the study, is that we have chosen not to take into account the history of fall incidents. A risk model for the prevention of recurrent falls in community-dwelling elderly of Stalenhoef et al., showed that two or more falls in the previous year is one of the main determinants of recurrent falls [2]. However, in real life, data about the history of fall incidents is not always available and the reliability of the outcome of the data would be questionable given the information bias in the fall incident reports in nursing home patients. The results of our study show that a clinical rule based only on electronically available data is insufficient to predict the fall risk in nursing home patients. However, the developed predictive clinical rule could serve as a basis for future research, since more data are made available electronically and this could mean that in the near future more risk factors can be added to the prediction model in order to optimize the model. Finally, the next step would be to perform a prospectively designed study for the validation of the predictive clinical rule in real life nursing home patients.

For peer review only

AUTHOR CONTRIBUTIONS

All authors have made substantial contributions to conception and design, acquisition of data, analysis and interpretation of data; They all have been involved in drafting the manuscript and revising it critically for important intellectual content; They all have given final approval of the version to be published; and they all agree to be accountable for all aspects of the work in ensuring that questions related to the accuracy or integrity of any part of the work are appropriately investigated and resolved.

Conception or design of the work

V Milosevic

AEMJH Linkens

KPGM Hurkens

DR Wong

B Winkens

BPC van Oijen

C Mestres-Gonzalvo

PHM van der Kuy

For peer review only - http://bmjopen.bmj.com/site/about/guidelines.xhtml

Data Collection

V Milosevic

PHM van der Kuy

Data analysis and interpretation

V Milosevic

C Mestres-Gonzalvo

BPC van Oijen

B Winkens

PHM van der Kuy

Drafting the article

V Milosevic

Critical revision of the article

AEMJH Linkens

KPGM Hurkens

B Winkens

BPC van Oijen

C Mestres-Gonzalvo

PHM van der Kuy

Final approval of the version to be published

V Milosevic

AEMJH Linkens

KPGM Hurkens

DR Wong

B Winkens

BPC van Oijen

C Mestres-Gonzalvo

PHM van der Kuy

For peer review only

ETHICAL CONSIDERATIONS (45)

The study was conducted retrospectively using existing data on patient characteristics, laboratory data, medication and reports on fall incidents from nursing home and/or hospital records. The study is approved (METCZ20180135) by the Medical Research Ethics Committee of Zuyderland Medical Centre and Zuyd University of Applied Sciences.

A patient consent form

As the data from the patients was anonymized after extraction no patient consent form was necessary according to the Medical Research Ethics Committee

A data sharing statement

The data underlying this article will be shared on reasonable request to the corresponding author.

For peer review only

For peer review only - http://bmjopen.bmj.com/site/about/guidelines.xhtml

References

[1] WHO Global report on falls prevention in older age, WHO ageing and life course, family and community health, World Health Organization 2007.

[2] Stalenhoef PA, Diederiks JP, Knottnerus JA, *et al.* A risk model for the prediction of recurrent falls in community-dwelling elderly: a prospective cohort study. *J Clin Epidemiol.* 2002 Nov;55(11):1088-94.

[3] Tinetti ME, Speechley M, Ginter SF. Risk factors for falls among elderly persons living in the community. *New England Journal of Medicine*. 1988;319:1701-1707.

[4] Downton JH, Andrews K, Prevalence, characteristics and factors associated with falls among the elderly living at home. *Aging*. 1991;3(3):219-28.

[5] Tinetti ME. Factors associated with serious injury during falls by ambulatory nursing home residents. *Journal of the American Geriatrics Society*. 1987;35:644-648.

[6] Nederlandse Vereniging voor Klinische Geriatrie (NVKG) Richtlijn Preventie van valincidenten bij ouderen (Guideline prevention of fall incidents in elderly patients, from the Dutch association of geriatrics), 2017. Available from: https://www.richtlijnendatabase.nl/richtlijn/preventie_van_valincidenten_bij_ouderen/startpagina_-_preventie_van_valincidenten.html, Feb 2018

[7] Helgadottir B, Laflamme L, Monarrez-Espino J, *et al*. Medication and fall injury in the elderly population: do individual demographics, health status and lifestyle matter? *BMC Geriatric* 2014;14:92

[8] Ham AC, Swart KM, Enneman AW., *et al*. Medication-related fall incidents in an older, ambulant population: I B-PROOF study. *Drugs Aging* 2014;31(12):917-27.

[9] Leipzig RM, Cumming RG, Tinetti ME. Drugs and falls in older people: a systematic review and meta-analysis: I. Psychotropic drugs. *J. Am. Geriatr. Soc.* 1999;47(1):30-9

[10] Leipzig RM, Cumming RG, Tinetti ME. Drugs and falls in older people: a systematic review and meta-analysis: II. Cardiac and analgesic drugs. *J. Am. Geriatr. Soc.* 1999; 47(1):40-50

[11] Woolcott JC, Richardson KJ, Wiens MO, *et al.* Meta-analysis of the impact of 9 medication classes on falls in elderly persons. *Arch Intern Med*. 2009;23;169(21):1952-60

[12] Janus SIM, Reinders GH, van Manen JG, *et al.* Psychotropic drug-related fall incidents in nursing home residents living in the eastern part of the Netherlands. *Drugs R D* (2017) 17:321-328

For peer review only

[13] Seppala LJ, Wermelink AMAT, de Vries M, *et al*. Fall-risk-increasing drugs: a systematic review and meta-analysis: II. Psychotropics. *J Am Med Dir Assoc.* 2018 Apr;19(4):371.e11-371.e17.

[14] Seppala LJ, van de Glind EMM, Daams JG, *et al.* Fall-risk-increasing drugs: a systematic review and meta-analysis: III. Others. *J Am Med Dir Assoc.* 2018 Apr;19(4):372.e1-372.e8.

[15] de Vries M, Seppala LJ, Daams JG, *et al.* Fall-risk-increasing drugs: a systematic review and meta-analysis: I. Cardiovascular drugs. *J Am Med Dir Assoc.* 2018 Apr;19(4):371.e1-371.e9.

[16] Bor A, Matuz M, Csatordai M, *et al.* Medication use and risk of falls among nursing home residents: a retrospective cohort study. *Int J Clin Pharm* (2017) 39:408-415

[17] Marcum ZA, Perera S, Thorpe JM, *et al.* Antidepressant use and recurrent falls in community-dwelling older adults: Findings from the health ABC study. *Ann Pharmacother.* 2016 July; 50(7):525-533

[18] Berry SD, Placide SG, Mostofsky E, *et al.* Antispychotic and benzodiazepine drug changes affect acute falls risk differently in the nursing home. *Journals of Gerontology: Med sciences,* 2016, Vol. 71, No. 2.

[19] Kuschel BM, Laflamme L, Möller J, The risk of fall injury in relation to commonly prescribed medications among older people-a Swedisch case-control study. *European Journal of Public Health*, Vol. 25, No. 3, 527-532.

[20] Maximos M, Chang F, Patel T, Risk of falls associated with antiepileptic drug use in ambulatory elderly populations: a systematic review. *Can Pharm J* (Ott) 2017; 150:101-111

[21] Matarese M, Ivziku D, Bartolozzi F, *et al.* Systematic review of fall risk screening tools for older patients in acute hospitals. *J Adv Nurs.* 2015 Jun; 71(6):1198-209

[22] Nunan S, Brown WC, Henwood T, *et al.* Fall risk assessment tools for use among older adults in long-term care settings: A systematic review of the literature. *Australas J Ageing.* 2018 Mar;37(1):23-33.

[23] Yazdani C, Hall S, Evaluation of the Medication fall risk score. *Am J Health-Syst Pharm.* 2017; 74:e32-9.

[24] Maurer MS, Burcham J, Cheng H, Diabetes Mellitus Is Associated With an Increased Risk of Falls in Elderly Residents of a Long-Term Care Facility. *Journal of Gerontology: MEDICAL SCIENCES*, 2005, Vol. 60A, No. 9, 1157–1162.

[25] Duh MS, Mody SH, Lefebvre P, *et al.*, Anaemia and the Risk of Injurious Falls in a Community-Dwelling Elderly Population. *Drugs Aging* 2008; 25 (4): 325-334

For peer review only - http://bmjopen.bmj.com/site/about/guidelines.xhtml

[26] Ambrosea AF, Geet P, Hausdorff JM, Risk factors for falls among older adults: A review of the literature. *Maturitas* 75 (2013) 51– 61.

[27] Dharmarajan TS, Avula S, Norkus EP, Anemia Increases Risk for Falls in Hospitalized Older Adults: An Evaluation of Falls in 362 Hospitalized, Ambulatory, Long-Term Care, and Community Patients. *Jamda* 2006.12.015

[28] Polinder-Bos HA, Emmelot-Vonk MH, Gansevoort RT, *et al.* High fall incidence and fracture rate in elderly dialysis patients. *The Netherlands Journal of Medicine*. Dec. 2014, Vol.72, No. 10

[29] Fehlberg EA, Lucero RJ, Weaver MT, Associations between hyponatraemia, volume depletion and the risk of falls in US hospitalized patients: a case–control study. *BMJ Open* 2017;7

[30] Jill Jin, Prevention of Falls in Older Adults, *JAMA*. 2018;319(16):1734

[31] Park H, Satoh H, Miki A, *et al.* Medications associated with falls in older people: systematic review of publications from a recent 5-year period. *Eur J Clin Pharmacol*. 2015 Dec;71(12):1429-40.

For peer review only

For peer review only - http://bmjopen.bmj.com/site/about/guidelines.xhtml

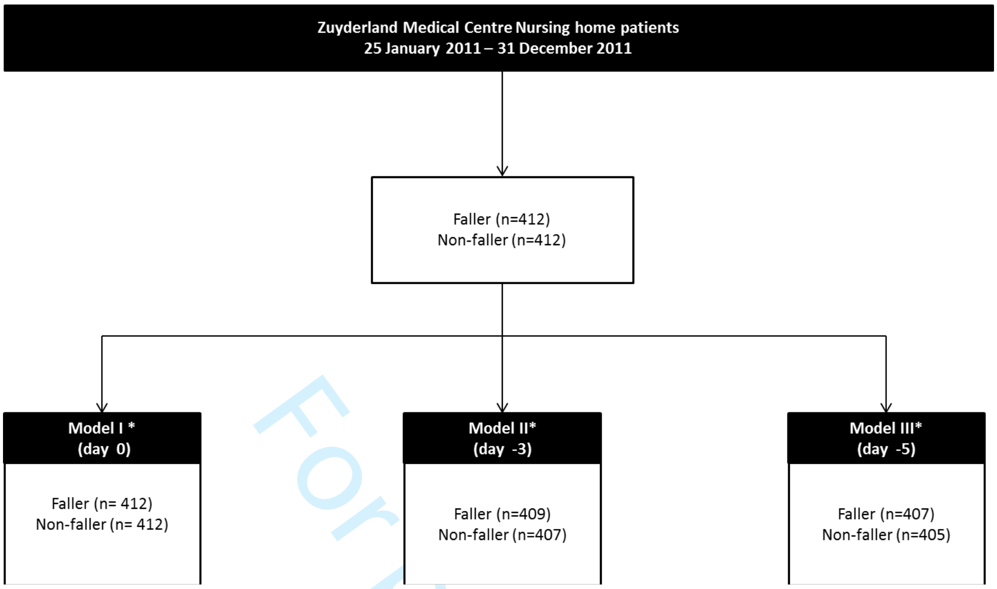

For peer review only

For peer review only - http://bmjopen.bmj.com/site/about/guidelines.xhtml

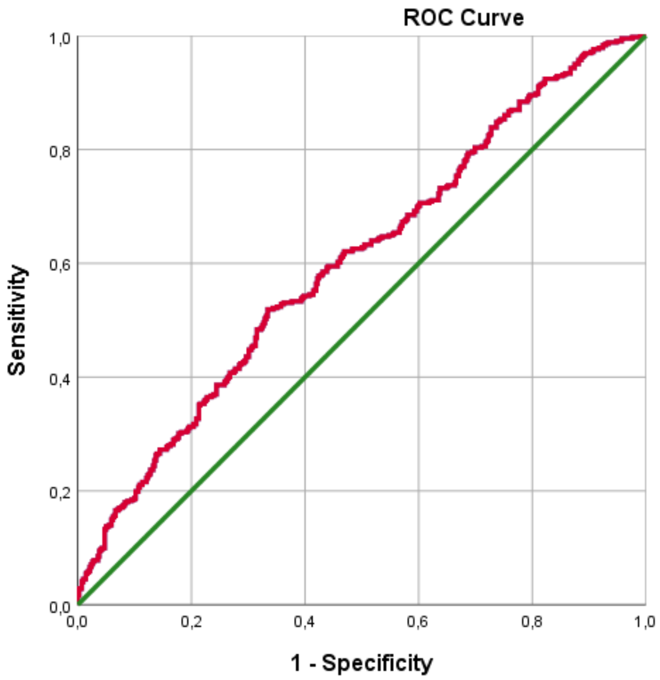

For peer review only - http://bmjopen.bmj.com/site/about/guidelines.xhtml

Supplementary Table 1a: Potential fall risk factors, Model I (day 0)

Risk factors	Mean (SD)	Frequency (number)	% of total	OR	95% C.I. OR		p-value
					Upper	Lower	
**Age (y)**	82.43 (8.46)	-	-	1.044	1.026	1.063	**.000** *
**Age(y) centred**	0.03 (8.46)	-	-	1.035	1.015	1.055	**.001** *
**Age(y) centred²**	71.57 (147.93)	-	-	.998	.996	.999	**.008** *
**Gender**	-	252 *M*, 572 *F*	30.58 *M*, 69.42 *F*	.561	.415	.758	**.000** *
**ATC unique**	8.49 (3.91)	-	-	1.035	.999	1.072	.059
**ATC unique≥5**		697	84.59	1.183	.810	1.729	.385
**ATC unique 0-5**		127	15.41	.845	.578	1.235	.385
**ATC unique 5- 10**		399	48.42	.864	.657	1.136	.296
**ATC unique 10-15**		226	27.43	1.103	.811	1.499	.531
**ATC unique ≥15**		72	8.74	1.640	1.000	2.690	**.050** *
**N06D**		97	11.77	6.057	3.474	10.558	**.000** *
**C01**		160	19.42	1.168	.827	1.651	.379
**N04A**		1	.12	*NA*	*NA*	*NA*	*NA*
**N06A**		253	30.70	1.394	1.035	1.878	**.029** *
**N03A**		75	9.10	.499	.304	.820	**.006** *
**R06A**		32	3.88	1.139	.561	2.313	.719
**N05A**		304	36.89	2.144	1.605	2.863	**.000** *
**C07**		199	24.15	.864	.628	1.190	.370
**S01ED**		24	2.91	1.414	0.621	3.222	0.409
**N05C**		280	33.98	1.417	1.060	1.894	**.019** *
**G04**		104	12.62	1.635	1.074	2.489	**.022** *
**C08**		80	9.71	.895	.564	1.420	.638
**C03**		281	34.10	1.177	.881	1.571	.270
**M03**		7	.85	.000	.000	*NA*	0.999
**C04AC**		0	.00	*NA*	*NA*	*NA*	*NA*
**C01C**		2	.24	1.000	.062	16.055	1.000
**N02A**		101	12.26	.589	.385	.903	**.015** *
**A02B**		318	38.59	.866	.654	1.147	.316
**C09**		211	25.61	1.039	.760	1.421	.811
**N07C**		20	2.43	1.000	.411	2.435	1.000
**N02B**		395	47.94	1.030	.783	1.355	.834
**N07AA**		3	.36	.498	.045	5.524	.571
**A10**		146	17.72	1.106	.772	1.582	.583
**C02**		6	.73	1.000	.200	4.988	1.000
**M01A**		54	6.55	1.083	.623	1.882	.778
**N07C**		20	2.43	1.000	.411	2.435	1.000
**N05B**		231	28.03	1.352	.996	1.835	**.053** *
**C10**		176	21.36	1.060	.759	1.479	.734
**R01BA**		0	.00	*NA*	*NA*	*NA*	*NA*
**C01A**		69	8.37	1.100	.671	1.804	.705
**C01B**		8	.97	1.000	.248	4.030	1.000
**C01D**		98	11.89	1.149	.753	1.754	.519
**N04**		37	4.49	.945	.488	1.828	.866
**N06DA**		41	4.98	5.217	2.284	11.917	**.000** *
**G04CA**		75	9.10	1.768	1.083	2.885	**.023** *

* statistically significant (p-value < 0.05)

NA = not available; SD = standard deviation; OR = Odds Ratio; C.I.= confidence interval; *M* = male, *F* = female

For peer review only

Supplementary Table 1b: Potential fall risk factors, Model II (day -3)

Risk factors	Mean (SD)	Frequency (number)	% of total	OR	95% C.I. OR		p-value
					Upper	Lower	
**Age (y)**	82.47 (8.39)	-	-	1.043	1.025	1.062	**.000** *
**Age(y) centred**	0.00 (8.39)	-	-	1.033	1.014	1.054	**.001** *
**Age(y) centred²**	70.35 (143.45)	-	-	.998	.996	.999	**.007** *
**Gender**	-	246 *M*, 570 *F*	30.15% *M*, 69.85% *F*	.546	.403	.740	**.000** *
**ATC unique**	8.46 (3.90)	-	-	1.029	.993	1.066	.115
**ATC unique≥5**		690	84.56	1.044	.714	1.527	.824
**ATC unique 0-5**		126	15.44	.958	.655	1.401	.824
**ATC unique 5- 10**		401	49.14	.872	.662	1.147	.328
**ATC unique 10-15**		221	27.08	1.057	.776	1.439	.726
**ATC unique ≥15**		68	8.33	1.467	.887	2.428	.136
**N06D**		97	11.89	6.553	3.707	11.584	**.000** *
**C01**		161	19.73	1.216	.861	1.718	.268
**N04A**		1	.12	*NA*	*NA*	*NA*	*NA*
**N06A**		253	31.00	1.386	1.028	1.868	**.032** *
**N03A**		75	9.19	.529	.323	.866	**.011** *
**R06A**		32	3.92	1.133	.558	2.301	.729
**N05A**		304	37.25	2.044	1.530	2.730	**.000** *
**C07**		199	24.39	.858	.623	1.182	.350
**S01ED**		24	2.94	1.408	.618	3.208	.416
**N05C**		277	33.95	1.365	1.020	1.828	**.036** *
**G04**		104	12.75	1.554	1.022	2.362	**.039** *
**C08**		81	9.93	.916	.579	1.450	.708
**C03**		278	34.07	1.132	.847	1.512	.404
**M03**		7	.86	.000	.000	*NA*	.999
**C04AC**		0	.00	*NA*	*NA*	*NA*	*NA*
**C01C**		2	.25	.994	.062	15.954	.996
**N02A**		95	11.64	.568	.366	.883	**.012** *
**A02B**		314	38.48	.840	.634	1.115	.228
**C09**		211	25.86	1.059	.774	1.449	.720
**N07C**		19	2.33	1.107	.444	2.764	.827
**N02B**		384	47.06	.933	.709	1.229	.624
**N07AA**		3	.37	.495	.045	5.489	.567
**A10**		143	17.52	1.119	.779	1.608	.541
**C02**		6	.74	.994	.199	4.958	.994
**M01A**		53	6.50	.955	.547	1.667	.871
**N07C**		19	2.33	1.107	.444	2.764	.827
**N05B**		229	28.06	1.380	1.015	1.877	**.040** *
**C10**		179	21.94	1.037	.745	1.446	.828
**R01BA**		0	.00	*NA*	*NA*	*NA*	*NA*
**C01A**		68	8.33	1.133	.688	1.865	.625
**C01B**		8	.98	.994	.247	4.006	.993
**C01D**		100	12.25	1.194	.784	1.816	.409
**N04**		37	4.53	.940	.486	1.818	.853
**N06DA**		40	4.90	6.071	2.519	14.628	**.000** *
**G04CA**		75	9.19	1.653	1.015	2.689	**.043** *

* statistically significant (p-value < 0.05)

NA = not available; SD = standard deviation; OR = Odds Ratio; C.I.= confidence interval; *M* = male, *F* = female

For peer review only

Supplementary Table 1c: Potential fall risk factors, Model III (day -5)

Risk factors	Mean (SD)	Frequency (number)	% of total	OR	95% C.I. OR		p-value
					Upper	Lower	
**Age (y)**	82.52 (8.36)	-	-	1.042	1.024	1.061	**.000 ***
**Age(y) centred**	0.00 (8.36)	-	-	1.033	1.013	1.053	**.001 ***
**Age(y) centred²**	69.75 (143.05)	-	-	.998	.996	.999	**.010 ***
**Gender**	-	243 *M*, 569 *F*	29.93 *M*, 70.07 *F*	.536	.395	.728	**.000 ***
**ATC unique**	8.44 (3.93)	-	-	1.023	.987	1.059	.214
**ATC unique≥5**		684	84.24	1.083	.742	1.581	.679
**ATC unique 0-5**		128	15.76	.923	.633	1.348	.679
**ATC unique 5- 10**		395	48.65	.906	.688	1.194	.484
**ATC unique 10-15**		223	27.46	1.083	.796	1.474	.613
**ATC unique ≥15**		66	8.13	1.296	.781	2.152	.316
**N06D**		94	11.58	6.833	3.801	12.285	**.000 ***
**C01**		160	19.70	1.198	.847	1.695	.306
**N04A**		1	.12	*NA*	*NA*	*NA*	*NA*
**N06A**		251	30.91	1.357	1.006	1.830	**.045 ***
**N03A**		74	9.11	.541	.330	.888	**.015 ***
**R06A**		30	3.69	.995	.480	2.063	.989
**N05A**		298	36.70	1.973	1.475	2.639	**.000 ***
**C07**		196	24.14	.868	.629	1.198	.391
**S01ED**		24	2.96	1.408	.618	3.208	.416
**N05C**		273	33.62	1.400	1.045	1.878	**.024 ***
**G04**		103	12.68	1.457	.958	2.215	.078
**C08**		78	9.61	.940	.589	1.499	.794
**C03**		277	34.11	1.120	.837	1.498	.446
**M03**		7	.86	.000	.000	*NA*	.999
**C04AC**		0	.00	*NA*	*NA*	*NA*	*NA*
**C01C**		2	.25	.994	.062	15.955	.996
**N02A**		91	11.21	.586	.374	.916	**.019 ***
**A02B**		312	38.42	.840	.633	1.114	.226
**C09**		209	25.74	1.060	.773	1.451	.719
**N07C**		19	2.34	1.107	.444	2.764	.827
**N02B**		383	47.17	.905	.687	1.194	.481
**N07AA**		3	.37	.495	.045	5.489	.567
**A10**		140	17.24	1.065	.739	1.534	.736
**C02**		6	.74	.994	.199	4.959	.994
**M01A**		54	6.65	.918	.528	1.597	.761
**N07C**		19	2.34	1.107	.444	2.764	.827
**N05B**		228	28.08	1.332	.980	1.812	.067
**C10**		175	21.55	1.038	.743	1.451	.826
**R01BA**		0	.00	*NA*	*NA*	*NA*	*NA*
**C01A**		68	8.37	1.133	.688	1.865	.625
**C01B**		8	.99	.994	.247	4.006	.993
**C01D**		99	12.19	1.168	.766	1.781	.469
**N04**		37	4.56	.940	.486	1.818	.853
**N06DA**		39	4.80	7.307	2.827	18.885	**.000 ***
**G04CA**		75	9.24	1.555	.958	2.524	.074

* statistically significant (p-value < 0.05)
NA = not available; SD = standard deviation; OR = Odds Ratio; C.I.= confidence interval; *M* = male, *F* = female

For peer review only

For peer review only - http://bmjopen.bmj.com/site/about/guidelines.xhtml

TRIPOD Checklist: Prediction Model Development

Section/Topic	Item	Checklist Item	Page
**Title and abstract**			
Title	1	Identify the study as developing and/or validating a multivariable prediction model, the target population, and the outcome to be predicted.	1
Abstract	2	Provide a summary of objectives, study design, setting, participants, sample size, predictors, outcome, statistical analysis, results, and conclusions.	3
**Introduction**			
Background and objectives	3a	Explain the medical context (including whether diagnostic or prognostic) and rationale for developing or validating the multivariable prediction model, including references to existing models.	5-6
	3b	Specify the objectives, including whether the study describes the development or validation of the model or both.	6
**Methods**			
Source of data	4a	Describe the study design or source of data (e.g., randomized trial, cohort, or registry data), separately for the development and validation data sets, if applicable.	7
	4b	Specify the key study dates, including start of accrual; end of accrual; and, if applicable, end of follow-up.	7
Participants	5a	Specify key elements of the study setting (e.g., primary care, secondary care, general population) including number and location of centres.	7
	5b	Describe eligibility criteria for participants.	7
	5c	Give details of treatments received, if relevant.	-
Outcome	6a	Clearly define the outcome that is predicted by the prediction model, including how and when assessed.	7
	6b	Report any actions to blind assessment of the outcome to be predicted.	-
Predictors	7a	Clearly define all predictors used in developing or validating the multivariable prediction model, including how and when they were measured.	7-8
	7b	Report any actions to blind assessment of predictors for the outcome and other predictors.	-
Sample size	8	Explain how the study size was arrived at.	8-9
Missing data	9	Describe how missing data were handled (e.g., complete-case analysis, single imputation, multiple imputation) with details of any imputation method.	13-16
Statistical analysis methods	10a	Describe how predictors were handled in the analyses.	7-9
	10b	Specify type of model, all model-building procedures (including any predictor selection), and method for internal validation.	7-9
	10d	Specify all measures used to assess model performance and, if relevant, to compare multiple models.	7-9
Risk groups	11	Provide details on how risk groups were created, if done.	-
**Results**			
Participants	13a	Describe the flow of participants through the study, including the number of participants with and without the outcome and, if applicable, a summary of the follow-up time. A diagram may be helpful.	figure 1
	13b	Describe the characteristics of the participants (basic demographics, clinical features, available predictors), including the number of participants with missing data for predictors and outcome.	table 1
Model development	14a	Specify the number of participants and outcome events in each analysis.	suplementary tables
	14b	If done, report the unadjusted association between each candidate predictor and outcome.	suplementary tables
Model specification	15a	Present the full prediction model to allow predictions for individuals (i.e., all regression coefficients, and model intercept or baseline survival at a given time point).	table 3
	15b	Explain how to the use the prediction model.	table 3
Model performance	16	Report performance measures (with CIs) for the prediction model.	table 4
**Discussion**			
Limitations	18	Discuss any limitations of the study (such as nonrepresentative sample, few events per predictor, missing data).	13-16
Interpretation	19b	Give an overall interpretation of the results, considering objectives, limitations, and results from similar studies, and other relevant evidence.	13-16
Implications	20	Discuss the potential clinical use of the model and implications for future research.	13-16
**Other information**			
Supplementary information	21	Provide information about the availability of supplementary resources, such as study protocol, Web calculator, and data sets.	-
Funding	22	Give the source of funding and the role of the funders for the present study.	4

We recommend using the TRIPOD Checklist in conjunction with the TRIPOD Explanation and Elaboration document.

For peer review only - http://bmjopen.bmj.com/site/about/guidelines.xhtml

BMJ Open

Fall Incidents in Nursing home patients: DEvelopment of a predictive clinical Rule (FINDER)

Journal:	*BMJ Open*
Manuscript ID	bmjopen-2020-042941.R1
Article Type:	Original research
Date Submitted by the Author:	01-Dec-2020

Complete List of Authors:	Milosevic, Vanja; Zuyderland Medical Centre Sittard-Geleen, Clinical Pharmacy, Pharmacology and Toxicology; Elkerliek Ziekenhuis, Clinical Pharmacy<br>Linkens, Aimee; Maastricht University Medical Centre+, Internal Medicine; Erasmus MC, University Medical Center Rotterdam<br>Winkens, Bjorn; Maastricht University, Methodology and Statistics<br>Hurkens, Kim; Zuyderland Medisch Centrum Heerlen, Geriatric Medicine, Department of Internal Medicine<br>Wong, Dennis; Zuyderland Medical Centre Sittard-Geleen, Clinical Pharmacy, Pharmacology and Toxicology<br>van Oijen, Brigit; Zuyderland, Clinical Pharmacy, Pharmacology and Toxicology<br>van der Kuy, Hugo; Erasmus MC, Clinical Pharmacy<br>Mestres-Gonzalvo, Carlota; Maastricht University Medical Centre+, Clinical Pharmacy and Toxicology

**Primary Subject Heading**:	Pharmacology and therapeutics
Secondary Subject Heading:	Geriatric medicine, Medical management
Keywords:	CLINICAL PHARMACOLOGY, GENERAL MEDICINE (see Internal Medicine), GERIATRIC MEDICINE

SCHOLARONE™
Manuscripts

I, the Submitting Author has the right to grant and does grant on behalf of all authors of the Work (as defined in the below author licence), an exclusive licence and/or a non-exclusive licence for contributions from authors who are: i) UK Crown employees; ii) where BMJ has agreed a CC-BY licence shall apply, and/or iii) in accordance with the terms applicable for US Federal Government officers or employees acting as part of their official duties; on a worldwide, perpetual, irrevocable, royalty-free basis to BMJ Publishing Group Ltd ("BMJ") its licensees and where the relevant Journal is co-owned by BMJ to the co-owners of the Journal, to publish the Work in this journal and any other BMJ products and to exploit all rights, as set out in our licence.

The Submitting Author accepts and understands that any supply made under these terms is made by BMJ to the Submitting Author unless you are acting as an employee on behalf of your employer or a postgraduate student of an affiliated institution which is paying any applicable article publishing charge ("APC") for Open Access articles. Where the Submitting Author wishes to make the Work available on an Open Access basis (and intends to pay the relevant APC), the terms of reuse of such Open Access shall be governed by a Creative Commons licence – details of these licences and which Creative Commons licence will apply to this Work are set out in our licence referred to above.

Other than as permitted in any relevant BMJ Author's Self Archiving Policies, I confirm this Work has not been accepted for publication elsewhere, is not being considered for publication elsewhere and does not duplicate material already published. I confirm all authors consent to publication of this Work and authorise the granting of this licence.

For peer review only

Title

Fall Incidents in Nursing home patients: DEvelopment of a predictive clinical Rule (FINDER)

Authors

Vanja Milosevic[1,2,6]

vmilosevic@elkerliek.nl

Aimee Linkens[3,6]

aimee.linkens@mumc.nl

Bjorn Winkens[4]

bjorn.winkens@maastrichtuniversity.nl

Kim Hurkens[5]

k.hurkens@zuyderland.nl

Dennis Wong[1]

d.wong@zuyderland.nl

Brigit van Oijen[1]

br.vanoijen@zuyderland.nl

Hugo van der Kuy[6]

h.vanderkuy@erasmusmc.nl

Carlota Mestres-Gonzalvo[7]

c.mestres@mumc.nl

[1]Department of Clinical Pharmacy, Pharmacology and Toxicology, Zuyderland Medical Centre, Dr. H van der Hoffplein 1, 6162 BG, Sittard-Geleen and Henri Dunantstraat 5, 6419 PC Heerlen, The Netherlands.

[2]Department of Clinical Pharmacy, Elkerliek Hospital, Wesselmanlaan 25, 5707 HA Helmond, The Netherlands.

[3]Department of Internal Medicine, Maastricht University Medical Centre+, P. Debyelaan 25, 6229 HX Maastricht, The Netherlands.

[4]Department of Methodology and Statistics, Faculty of Health, Medicine and Life Sciences, Maastricht University, P. Debyeplein 1, 6229 HA Maastricht, The Netherlands.

[5]Department of Internal Medicine sect. of Geriatric Medicine, Zuyderland Medical Centre, Dr. H van der Hoffplein 1, 6162 BG, Sittard-Geleen and Henri Dunantstraat 5, 6419 PC Heerlen, The Netherlands.

For peer review only

For peer review only - http://bmjopen.bmj.com/site/about/guidelines.xhtml

[6]Erasmus MC, University Medical Center Rotterdam, Department of Hospital Pharmacy, Postbus 2040, 3000 CA Rotterdam, The Netherlands.

[7]Department of Clinical Pharmacy and Toxicology, Maastricht University Medical Centre+, P. Debyelaan 25, 6229 HX Maastricht, The Netherlands.

Corresponding Author:

Prof. dr. Hugo van der Kuy

Department of Clinical Pharmacy

Erasmus Medical Centre

Postbus 2040

3000 CA Rotterdam

The Netherlands

Email: h.vanderkuy@erasmusmc.nl

Keywords

Fall risk, Decision Support System, nursing home patients, ROC Curve, Logistic Models.

Word count: 3.808 (without abstract, strength and limitations, literature, figures and tables)

Number of tables: 3

Number of figures: 2

Number of supplementary tables: 3 (1a,b and c)

Number of supplementary texts: 0

For peer review only

For peer review only - http://bmjopen.bmj.com/site/about/guidelines.xhtml

ABSTRACT (298)

Objectives: To develop (part I) and validate (part II) an electronic fall risk clinical rule (CR) to identify nursing home patients (NH-patients) at risk for a fall incident.

Design: Observational, retrospective database study.

Setting: Nursing homes

Participants: A total of 1668 (824 in part I, 844 in part II) NH-patients from The Netherlands were included. Data of participants from part I were excluded in part II.

Primary and secondary outcome measures: Development and validation of a fall risk CR in NH-patients. Logistic regression analysis was conducted to identify the fall risk-variables in part I. With these, three CRs were developed (i.e. at the day of the fall incident and three days and five days prior to the fall incident). The overall prediction quality of the CRs were assessed using the Area Under the Receiver Operating Characteristics (AUROC), and a cut-off value was determined for the predicted risk ensuring a sensitivity ≥ 0.85. Finally, one CR was chosen and validated in part II using a new retrospective dataset.

Results: Eleven fall risk-variables were identified in part I. The AUROCs of the three CRs form part I were similar: the AUROC for model I, II and III were 0.714 (95% CI 0.679 to 0.748), 0.715 (95%CI 0.680 to 0.750) and 0.709 (95%CI 0.674 to 0.744), respectively. Model III (i.e. 5 days prior to the fall incident) was chosen for validation in part II. The validated AUROC of the CR, obtained in part II, was 0.603 (95%CI 0.565 to 0.641) with a sensitivity of 83.41% (95% CI 79.44% to 86.76%) and a specificity of 27.25%(95% CI 23.11% to 31.81%).

Conclusion: Medication data and patient characteristics alone are not sufficient enough to develop a successful CR with a high sensitivity and specificity to predict fall risk in NH-patients.

Trial registration: not available

For peer review only

STRENGHTS AND LIMITATIONS OF THIS STUDY

- Currently there is no golden standard in the Netherlands regarding the use of a specific tool in the prevention of fall risk in NH-patients.
- This study was based on electronic data and variables that are currently available in daily practice and therefore easy to implement. However, fall risk factors that were not available electronically are lacking.

For peer review only - http://bmjopen.bmj.com/site/about/guidelines.xhtml

- The CR was developed and validated using two different retrospective data sets of the same nursing homes and therefore lacks a prospective validation in a different nursing home setting.

FUNDING

This research received no specific grant from any funding agency in the public, commercial or not-for-profit sectors.

Competing Interests

All authors have declared that no competing interests exist.

Abbreviations

Clinical rule (CR), nursing home patients (NH-patients), Clinical Decision Support System (CDSS), fall risk increasing drugs (FRIDs), receiver operating characteristics (ROC), area under the receiver operating characteristics (AUROC),

For peer review only

For peer review only - http://bmjopen.bmj.com/site/about/guidelines.xhtml

INTRODUCTION / BACKGROUND (715)

Worldwide there are 646,000 falls every year which result in death, making falls the second leading cause of unintended death [1]. Falls are common in elderly people aged 65 years and over. According to the World Health Association, 28-35% of the elderly fall each year [1]. This percentage increases with age (e.g. 32-42% in the age group of 70 years and older) as does the fatal falls rate [2-4]. However, not only age seems to be related to the risk of falls, the frailty level is also a risk factor. For example, elderly who live in nursing homes have higher falling rates compared to those living in the community [5]. The incidence of falls in NH-patients ranges from 30 to 50% per year [1,6].
The ageing population challenges us with the question: how to prevent falls?

There are four different dimensions involved in fall incidents: biological (e.g. race, gender, age, (multi)-morbidity), environmental (e.g. building design, insufficient light), socio-economic (e.g. inadequate housing, limited access to social services and health), and behavioural factors (e.g. polypharmacy, inappropriate foot ware) [1].

Focusing on behavioural aspects, specifically on medication, several studies have been conducted to investigate the effect of medication on fall risk [1,7-20]. These studies have shown that the risk of falls resulting in injuries and/or death, increases with increasing numbers of drugs used in the elderly [7]. Other studies investigated the association of different drug classes with an increased risk of falls [11-20]. Examples of identified drug classes with an increased fall risk are anti-arrhythmic drugs, antidepressants, psychotropic drugs, and diuretics [1,8-11].

Different strategies and tools to prevent fall incidents are developed [21]. However, given the multifactorial aspect and complexity of variables of fall incidents, it is extremely difficult to obtain a tool with a high sensitivity and specificity, and one that is easy to implement in practice [1,21,22].

The current tools differ in number and type of fall risk factors included (e.g. multifactorial assessment tools, functional mobility or medication related assessment tools). Also, the applicability is often restricted to one specific setting or subpopulation (e.g. hospital, nursing home, ambulatory). In The Netherlands, there is still no recommendation for one specific tool in the prevention of fall risk in NH-patients, leading to local differences regarding fall risk management.

For peer review only - http://bmjopen.bmj.com/site/about/guidelines.xhtml

Of the most commonly used tools for the prediction of fall risk (i.e. Hendrich Fall Risk Model I and II (HFRM I and II), St. Thomas's risk assessment tool in falling elderly inpatients (STRATIFY) and the Morse Fall Scale (MFS)), only the HFRM II tool makes use of information regarding the use of anti-epileptics and benzodiazepines. Both MFS and STRATIFY have no medication related fall risk factors included in the tool [6,23].

Nunan et al. [22], conducted a systematic review regarding fall risk assessment tools in the long-term care setting. In general, the following remarks can be made on current tools for prevention of fall incidents in the long-term care setting: they are either not validated, or have a low sensitivity and/or specificity (lower than 70%), and/or the practical applicability is below the desired level [22]. Therefore, there is still room for improvement.

Given the multifactorial aspect of fall incidents, a tool that combines all of the above-described dimensions (i.e. biological, environmental, socio-economic and behavioural) involved in fall incidents, would probably give the most accurate results. Currently there is no such tool available.

Our goal is to focus on all of the electronically available data of interest in fall incidents, aiming to combine as many different dimensions involved in fall incidents as possible. Thereby we will include age, gender, the presence of specific medication (expressed as ATC-codes i.e. codes from the Anatomical Therapeutic Chemical classification system), the number of unique ATC-codes and laboratory parameters. The specific ATC-codes and laboratory parameters that will be included in this study, are those from which there is evidence from other studies that there is a (possible) association with the fall risk.

With a Clinical Decision Support System (CDSS), one is able to combine different data, such as patient characteristics, laboratory and medication data, with the goal to generate specific alerts per patient. Developing and implementing a predictive CR for fall risk in an electronic CDSS could therefore be helpful for healthcare professionals in order to prevent falls in those who are at increased risk.

Objective (35)

The objective of this study is to develop (part I) and validate (part II) a fall risk CR that can be used in an electronic CDSS to identify NH-patients at risk for a fall incident.

For peer review only

For peer review only - http://bmjopen.bmj.com/site/about/guidelines.xhtml

METHODS (1091)

Design (61)

An observational retrospective database study was conducted in two parts. In part I, a predictive fall risk CR for implementation in an electronic CDSS was developed in a dataset of NH-patients. In part II, the developed CR was retrospectively validated in a different dataset of NH-patients.

For both parts of the study, data from NH-patients from Sittard-Geleen, The Netherlands, were used.

Patients selection (42)

Patients living in nursing homes were selected. The population consists of both somatic and non-somatic patients. For part I of the study, no exclusion criteria were used. In part II of the study, participants (fallers and non-fallers) from part I were excluded.

Outcome (125)

The outcome that is predicted by the fall risk CR is a fall.

In part I, NH-patients who have fallen at least once within the period of January 25th, 2011 until December 31th, 2011, were defined as *fallers*. NH-patients who have not fallen in the same period, were defined as *non-fallers*. In part II the same design was used as in part I. Part II was conducted in a new dataset of NH-patients in the period of January 25th, 2016 until December 31th, 2016.

In both parts of the study, the date of the first fall of the faller within the study period was set as the reference date. On this specific reference date, a control patient was selected at random from the non-fallers group.

Potential predictors (235)

Hospital and nursing home electronic systems were used to extract data on patient characteristics (i.e. age and gender), laboratory data, medication (number and type of

For peer review only

For peer review only - http://bmjopen.bmj.com/site/about/guidelines.xhtml

medicines), and reports on fall incidents. More specifically, data on medication were collected at the reference date and 3 and 5 days prior to the reference date, while laboratory data of approximately up to 3 months prior to the reference date were retrieved.

The laboratory parameters intended for analysis, are laboratory parameters known from literature to be associated with fall risk: albumin, sodium, potassium, creatinine, kidney clearance (MDRD), glucose and hemoglobin [24-29]. Regarding the medication data, the patients medication list was screened for presence of 35 different ATC-codes all of which are shown to have a (possible) association with fall risk [6,8-20]. Also the number of unique ATC-codes was collected.

Finally, electronically available patient characteristics, such as age and gender at time of the reference date, as well as the reports on fall incidents (date of the report), were obtained.

The described above dataset for part I was analysed in order to develop a predictive fall risk CR for an electronic CDSS based on relevant risk factors for the prediction of the fall risk in NH-patients. In part II, the developed CR was validated by determining its specificity and sensitivity using a new dataset of Zuyderland MC NH-patients obtained in the period of January 25th, 2016 until December 31th, 2016.

Statistical analysis (361)

Statistical analyses were performed using IBM SPSS Statistics for Windows (version 21.0. Armonk, NY: IBM Corp.). Two-sided p-values smaller than or equal to 0.05 were considered statistically significant.

For part I of the study, logistic regression analysis was used to identify statistically significant risk factors for the prediction of the fall risk, where variables significantly related to the outcome were included in the multiple logistic regression model. Three predictive models were created as medication data were collected at three different moments, i.e. at the time of the reference date (model I), 3 days prior to the reference date (model II), and 5 days prior to the reference date (model III). As laboratory data (collected up to approximately 3 months prior to the reference date) was only available in 411 out of 824 NH-patients (49.9%), the models were only based on medication data (specific ATC codes), age and gender. One model was eventually chosen for development and validation of the predictive CR.

For peer review only - http://bmjopen.bmj.com/site/about/guidelines.xhtml
For peer review only

Linearity assumption was checked for numerical variables by centering these variables and adding the quadratic centered term to the model. If this quadratic term was significant, the linear as well as quadratic term for this variable were included in the multiple regression model. The predictive CR (multiple linear regression model) was then used to obtain a predictive score which quantifies the estimated fall risk of the individual nursing home patient according to his/her specific risk factors. The bootstrap method was used (1000 bootstrap datasets) to obtain a shrinkage factor in order to correct for over-fitting. The overall predictive quality of the CR was determined by the c-statistic, i.e. the area under the receiver operating characteristics (AUROC) curve. Additionally, an optimal cut-off point for the estimated fall risk was obtained by two criteria: A) maximizing the Youden's J-statistic, i.e. sensitivity+specificity-1, and B) maximizing specificity where the sensitivity is at least 85%.

The predictive CR was applied to the dataset from part II of the study, where the optimal cut-off value obtained from part I was then used to determine the validated prognostic values of the CR. To obtain 95% confidence intervals around these prognostic values an online calculator (http://vassarstats.net/clin1.html) was used.

Sample size calculation (232)

The sample size was estimated for a CR that could consist of 30-40 risk factors. This number of risk factors was based on the number of different ATC-codes (fall risk increasing drugs (FRIDs), known from literature), use of polypharmacy (≥5 different ATC-codes in chronic use), the number of relevant laboratory data, and the patient characteristics age and gender [6,8-20,24-29]. The multiple logistic regression model requires at least 10 fallers for each individual risk factor. In order to obtain a representative incidence of fallers (about 50% per year) in the study population, also 10 non-fallers are needed per individual risk factor. This means that a total of approximately 800 NH-patients (at least 400 fallers and 400 non-fallers) were needed to carry out part I of the study, and another 800 NH-patients were needed for part II of the study (at least 400 fallers and 400 non-fallers).

These numbers would be large enough to ensure precise estimates of sensitivity and specificity of the predictive CR. More specifically, the width of the 95% confidence interval (CI) of sensitivity and specificity of the predictive CR would be maximally equal to $2*1.96*0.5/\sqrt{n} = 1.96/\sqrt{n}$, where n equals the number of fallers for sensitivity and number of non-fallers for specificity. Therefore, with at least 400 fallers and 400 non-fallers, the 95%CI

For peer review only

For peer review only - http://bmjopen.bmj.com/site/about/guidelines.xhtml

of sensitivity and specificity of the predictive CR would not be wider than 0.10 (1.96/√400 = 0.098).

Patient and Public Involvement (20)

Patients or the public were not involved in the design, or conduct, or reporting, or dissemination plans of our research.

RESULTS (342)

Participants

A total of 824 NH-patients (412 fallers and 412 non-fallers) in the time period of January 25th, 2011 up to December 31th, 2011 were included in part I of the study (Figure 1). Characteristics of the included NH-patients for part I of the study are shown in Table 1.

Table 1: Patient characteristics in part I of the study

	**Model I (day 0)**			**Model II (day -3)**			**Model III (day -5)**		
	Faller (n=412)	Non-faller (n=412)	Total (n=824)	Faller (n=409)	Non-faller (n=407)	Total (n=816)	Faller (n=407)	Non-faller (n=405)	Total (n=812)
**Mean Age ± SD* (y)**	83.91 ±6,87	80.96 ±9,59	82.43 ±8,46	83.89 ±6,86	81.05 ±9,49	82.47 ±8,39	83.90 ±6,88	81.13 ±9,42	82.52 ±8,36
**Male**	151 (36.7%)	101 (24.5%)	252 (30.6%)	149 (36.4%)	97 (23.8%)	246 (30.1%)	148 (36.4%)	95 (23.5%)	243 (29.9%)
**Female**	261 (63.3%)	311 (75.5%)	572 (69.4%)	260 (63.6%)	310 (76.2%)	570 (69,9%)	259 (63.6%)	310 (76.5%)	569 (70.1%)

*SD= standard deviation; y =years

Model development and specification

Table 2 shows the variables that were selected from literature and included for analysis in part I of the study.

For peer review only

For peer review only - http://bmjopen.bmj.com/site/about/guidelines.xhtml

Table 2: Fall risk variables

Variable	Description
**Polyfarmacy**	
ATCunique	Number of unique ATC codes
ATCunique ≥5	Presence of 5 or more unique ATC codes (yes=1, no=0)
ATCunique 0 - 5	Presence of 0-5 unique ATC codes (yes=1, no=0)
ATCunique 5 - 10	Presence of 5-10 unique ATC codes (yes=1, no=0)
ATCunique10 - 15	Presence of 10-15 unique ATC codes (yes=1, no=0)
ATCunique ≥ 15	Presence of 15 or more unique ATC codes (yes=1, no=0)
**Patient characteristics**	
Gender	Male / Female
Age	Age in years
Age_c	Age in years, centered
Age_c2	Age in years, centered quadratic term
**Presence of specific ATC codes (yes=1, no=0)**	
A02B	DRUGS FOR PEPTIC ULCER AND GASTRO-OESOPHAGEAL REFLUX DISEASE (GORD)
A10	DRUGS USED IN DIABETES
C01	CARDIAC THERAPY
C01A	CARDIAC GLYCOSIDES
C01B	ANTIARRHYTHMICS, CLASS I AND III
C01C	CARDIAC STIMULANTS EXCL. CARDIAC GLYCOSIDES
C01D	VASODILATORS USED IN CARDIAC DISEASES
C02	ANTIHYPERTENSIVES
C03	DIURETICS (cardiovascular system)
C04AC	Nicotinic acid and derivatives (peripheral vasodilators)
C07	BETA BLOCKING AGENTS (cardiovascular system)
C08	CALCIUM CHANNEL BLOCKERS (cardiovascular system)
C09	AGENTS ACTING ON THE RENIN-ANGIOTENSIN SYSTEM
C10	LIPID MODIFYING AGENTS
G04	UROLOGICALS
G04CA	Alpha-adrenoreceptor antagonists (urologicals)
M01A	ANTIINFLAMMATORY AND ANTIRHEUMATIC PRODUCTS, NON-STEROIDS
M03	MUSCLE RELAXANTS
N02A	OPIOIDS (analgetics)
N02B	OTHER ANALGESICS AND ANTIPYRETICS
N03A	ANTIEPILEPTICS
N04	ANTI-PARKINSON DRUGS
N04A	ANTICHOLINERGIC AGENTS (Anti-parkinson drugs)
N05A	ANTIPSYCHOTICS
N05B	ANXIOLYTICS
N05C	HYPNOTICS AND SEDATIVES
N06A	ANTIDEPRESSANTS
N06D	ANTI-DEMENTIA DRUGS
N06DA	Anticholinesterases (psychoanaleptics)
N07AA	Anticholinesterases (parasympathomimetics)
N07C	ANTIVERTIGO PREPARATIONS
R01BA	Sympathomimetics (respiratory system)
R06A	ANTIHISTAMINES FOR SYSTEMIC USE
S01ED	Beta blocking agents (ophthalmologicals)

For peer review only

For peer review only - http://bmjopen.bmj.com/site/about/guidelines.xhtml

From the 44 variables studied in total (Table 2), only 11 variables were found to be significantly related to fall risk (supplementary table 1a,b,c). The multiple logistic regression models consisting of these 11 variables, were applied to the data. The AUROC for model I, II and III were 0.714 (95% CI 0.679-0.748), 0.715 (95%CI 0.680-0.750) and 0.709 (95%CI 0.674-0.744), respectively.

For the development of the predictive CR, model III (5 days before reference day) was chosen, since the predictive quality of models I, II and III were similar. Based on the significant fall risk variables, and after correcting for over-fitting (shrinkage factor = 0.8979), the following predictive CR was developed to calculate the fall risk (Table 3).

Table 3: Fall risk predictive algorithm (*CR*) for model III (5 days prior to reference day).

Fall risk predictive CR (model III) in NH-patients:
Fall risk = 1/(1+Exp(-Linear predictor).

Linear predictor = 0.7799 + *0.8979**(-0.6802*V1 + 0.0460*V2- -0.0016*V3+ 1.6442*V4 + 0.2664*V5 -0.4763*V6 + 0.4267*V7 + 0.2368*V8 + 0.0001*V9 -0.3780*V10+0.2927*V11)
(shrinkage factor =0.8979)

Predictive fall risk factors:

V1	Gender (1 = male, 2 = female)
V2	Age_c = age -82,52 (age centered, linear term)
V3	Age_c2 = $(age\_c)^2$ (age centered, quadratic term)
V4	Presence of medicines with ATC code NO6D (yes=1, no=0)
V5	Presence of medicines with ATC code N06A (yes=1, no=0)
V6	Presence of medicines with ATC code N03A (yes=1, no=0)
V7	Presence of medicines with ATC code N05A (yes=1, no=0)
V8	Presence of medicines with ATC code N05C (yes=1, no=0)
V9	Presence of medicines with ATC code G04 (yes=1, no=0)
V10	Presence of medicines with ATC code N02A (yes=1, no=0)
V11	Presence of medicines with ATC code N05B (yes=1, no=0)

The optimal cut-off value for fall risk, using Youden's J-statistic, was 0.5726, resulting in a sensitivity of 46.9% and a specificity of 81.7%. If the optimal cut-off value was based on the second criterion, where the sensitivity should at least be 85.0%, then the associated cut-off value was 0.4303, resulting in a sensitivity of 85.0% and a specificity of 40.7%.

For peer review only

Model performance

In part II of the study, the developed CR was validated in a cohort of 844 NH-patients (422 fallers and 422 non-fallers) in the time period of January 25th, 2016 to December 31th, 2016. Applying the predictive CR on this cohort of NH-patients resulted in an AUROC of 0.603 (95%CI 0.565-0.641) (Figure 2). If the cut-off value for the fall risk was based on the second criterion (sensitivity ≥ 85%), then the validated sensitivity and specificity were 83.4% (95% CI 79.4% - 86.8%) and 27.3% (95% CI 23.1%-31.8%), respectively.

DISCUSSION AND CONCLUSION (1625)

A CR for NH-patients was developed and validated. This resulted in a CR which can be used to predict the fall risk 5 days prior to the fall incident, based on 11 variables, which were found to be significantly related to fall risk as shown in part I of the study. After identification of the 11 variables, three prediction models were compared: a fall risk prediction model at the reference date, 3 days prior to the reference date and 5 days prior to the reference date. Since the AUROC of all three models was similar, we choose to validate (in part II) the model to predict the fall incidence 5 days prior to the actual event. This decision was made because in daily practice, it is preferred to predict the fall incident as early as possible in order to take the necessary actions.

Since fall incidents are a massive problem in society and often result in a hospital admission or high mortality rates, we have chosen to set the sensitivity of the prediction model at 85.0%. A sensitivity of 85.0% results in missing less fall incidents.

A critical remark is that validation of the CR (part II of the study) resulted in 83.4% sensitivity and 27.3% specificity. A specificity of 27.3% means approximately 73% of the patients who would not fall within 5 days, would incorrectly be classified as having an increased fall risk. Since NH-patients are vulnerable, it may be useful to review the medication regardless of the fall risk. Also, the actions that are taken in fall risk prevention are mostly not drug related, but include e.g. exercise therapy, psychological health, vision, environmental conditions, diet and nutrition and sometimes may include medication [30]. In addition, it is still up to the healthcare professional to review per individual whether actions are needed to be taken to prevent falls and if so, which actions need to be taken, customized to the subject's needs.

The low specificity after validation of the CR, is probably due to the differences between both datasets (part I and II of the study). The ratio between males and females is different

For peer review only

For peer review only - http://bmjopen.bmj.com/site/about/guidelines.xhtml

between the two datasets, also the presence of specific ATC codes is different. In addition, the medication dosage and the duration of use is not included in the CR, which may vary between the two datasets. Compared to commonly used fall risk tools, the CR developed in this study, has a higher specificity than the HFRM I tool (sensitivity 97%, specificity 9%) and a higher sensitivity than the STRATIFY tool (sensitivity 63%, specificity 71%) [21]. However, the CR has a lower specificity compared to the STRATIFY tool, and a lower sensitivity compared to HFRM I [21]. When compared to HFRM II (sensitivity 92%, specificity 37%), both sensitivity and specificity are lower [21]. It is important to mention, however, that sensitivity and specificity of the STRATIFY, HFRM I and HFRM II tools are evaluated in older patients in acute care hospitals. Nunan et al. [22] conducted a systematic review in the long-term care setting. The sensitivity and specificity scores ranged from 50% to 91%, and from 32% to 90% respectively, for eight different multifactorial assessment tools (MATs). One of these MATs was the STRATIFY tool. In the long-term care setting, the STRATIFY tool showed a sensitivity of 50% (vs. 63% in hospital setting) and a specificity of 76% (vs. 71% in hospital setting) [22]. The HFRM I and II tools were not included in the study. The MAT tool with the highest sensitivity in the long-term care setting, was the Downton Index (sensitivity 91%, specificity 39%). The highest specificity was achieved with the Peninsula Health Falls Risk Assessment Tool (PHFRAT) (specificity 90%, sensitivity 58%) [22], however, this tool was validated retrospectively. The CR developed in our study has lower sensitivity and specificity scores compared to both the Downton Index and the PHFRAT.

The rather low AUROC for the prediction model in our study, means that the model is unsuccessful at distinguishing between classes (here: fallers and non-fallers). The optimal AUROC value would be 1.0. In the current study, we found an AUROC of 0.603 (95%CI 0.565-0.641). The low AUROC is probably due to the fact that we choose to base the CR on electronically available data only, in order to make it more suitable for implementation in daily practice. Making this choice, also means that it is not possible to implement important risk factors, like frailty, in the CR. The low AUROC therefore confirms the complex and multifactorial aspects of the risk of fall incidents, since only a few (i.e. gender, age and medication related data) of the fall risk factors were included in the predictive CR.

Besides the included fall risk factors in the developed predictive CR, the intention was to include laboratory data as well, in order to get the most out of the electronically available data. Given the fact that laboratory checks are not performed on daily basis in the nursing home setting, we choose to collect laboratory data up to three months prior to the date of the

For peer review only - http://bmjopen.bmj.com/site/about/guidelines.xhtml

For peer review only

fall incident. We intended to consider only the most recent known laboratory value of the specific laboratory parameter in the analysis. However, only in approximately 50% of the NH-patients was laboratory data available, for at least one of the laboratory parameters intended for analysis. Taking these data into account in the analysis, would result in a substantial loss of power. Therefore, no association of laboratory data with fall risk was studied.

Regarding the medication data, the presence of specific ATC-codes in the NH-patients medication list was checked. This was carried out at three specific moments: at the day of the fall incident, three days prior to the fall incident and five days prior to the fall incident. Since the association between the number of medications, types of medication, and the risk of falls was studied, medication in use close to the time of the fall incident was collected. It is difficult to define what the exact time period should be, because the effect of a specific medicine on a fall is possibly related to the half-life of the drug; However, it could also result from drug-drug interactions. The half-life of the drug and the presence or absence of drug-drug interactions is different for every patient, therefore, we chose to compare the association at the time of the fall incident and three and five days prior to the fall incident.

Regarding the results on the association of specific ATC codes with fall risk, we only found NO6D (anti-dementia drugs), N06A (antidepressants), N03A (antiepileptics), N05A (antipsychotics), N05C (hypnotics and sedatives), G04 (urologicals), N02A (opioids) and N05B (anxiolytics) to be significantly associated. The other ATC codes were not significant despite their found association in other studies [6,8-20]. However, this could also be due to the fact that some of the ATC codes were not present at all or only for a small group of NH-patients. Furthermore, in our study, polypharmacy was not statistically significantly related to fall risk. Although there are studies that show that polypharmacy increases the risk of falls, a meta-analysis showed that from the 19 studies that investigated the association of polypharmacy in older people with the risk of falls, only 6 studies reported that polypharmacy increases the risk of falling also the odds ratio reporting this association was nearing 1.0 [31]. The question therefore arises whether polypharmacy itself, or only the number of fall risk increasing drugs is a fall risk increasing variable. Further research is needed to investigate this.

The study has a few limitations. First of all, in this study the model (CR) was based on a fall incidence of 50% per year. If this incidence is incorrect, the predicted probabilities of this model are not correct either and we could only assess the C-statistic (AUROC). The conclusion that this model is not clinically useful still holds, as it was based on this C-

For peer review only

For peer review only - http://bmjopen.bmj.com/site/about/guidelines.xhtml

statistic. Another limitation of the study, is that the dataset of the included NH-patients consisted of fallers and non-fallers. The fallers were classified as such based on fall reports. It is unknown whether these fall reports are complete and which definition of a fall incident is used for reporting. It is also known that fall reports which are not fully completed, are not reported. In other words, the number of actual fallers is probably underestimated. Also, for practical reasons we did not collect information on patient characteristics such as mobility device use or the type of disability, etc.

Furthermore, we have chosen not to take into account the history of fall incidents. A risk model for the prevention of recurrent falls in community-dwelling elderly of Stalenhoef et al., showed that two or more falls in the previous year is one of the main determinants of recurrent falls [2]. However, in real life, data about the history of fall incidents is not always available and the reliability of the outcome of the data would be questionable given the information bias in fall incident reports in NH-patients.

The results of our study show that a CR based only on electronically available data is insufficient to predict the fall risk in NH-patients. However, the developed predictive CR could serve as a basis for future research, since more data are made available electronically and this could mean that in the near future more risk factors can be added to the prediction model in order to optimize the model. We would recommend for future research to include medication dosage and duration of medication use in the prediction model as well as frailty and history of falls. Finally, the next step would be to perform a prospectively designed study for validation of the predictive CR in real life NH-patients.

For peer review only

AUTHOR CONTRIBUTIONS

All authors have made substantial contributions to conception and design, acquisition of data, analysis and interpretation of data; They all have been involved in drafting the manuscript and revising it critically for important intellectual content; They all have given final approval of the version to be published; and they all agree to be accountable for all aspects of the work in ensuring that questions related to the accuracy or integrity of any part of the work are appropriately investigated and resolved.

Conception or design of the work

V Milosevic

AEMJH Linkens

KPGM Hurkens
DR Wong
B Winkens
BPC van Oijen
C Mestres-Gonzalvo
PHM van der Kuy

Data Collection

V Milosevic
PHM van der Kuy

Data analysis and interpretation

V Milosevic
C Mestres-Gonzalvo
BPC van Oijen
B Winkens
PHM van der Kuy

Drafting the article

V Milosevic

Critical revision of the article

AEMJH Linkens
KPGM Hurkens
B Winkens
BPC van Oijen
C Mestres-Gonzalvo
PHM van der Kuy

Final approval of the version to be published

V Milosevic
AEMJH Linkens

For peer review only

KPGM Hurkens
DR Wong
B Winkens
BPC van Oijen
C Mestres-Gonzalvo
PHM van der Kuy

For peer review only

For peer review only - http://bmjopen.bmj.com/site/about/guidelines.xhtml

ETHICAL CONSIDERATIONS (45)

The study was conducted retrospectively using existing data on patient characteristics, laboratory data, medication and reports on fall incidents from nursing home and/or hospital records. The study is approved (METCZ20180135) by the Medical Research Ethics Committee of Zuyderland Medical Centre and Zuyd University of Applied Sciences.

A patient consent form

As the data from the patients was anonymized after extraction no patient consent form was necessary according to the Medical Research Ethics Committee

A data sharing statement

The data underlying this article will be shared on reasonable request to the corresponding author.

For peer review only

For peer review only - http://bmjopen.bmj.com/site/about/guidelines.xhtml

References

[1] WHO Global report on falls prevention in older age, WHO ageing and life course, family and community health, World Health Organization 2007.

[2] Stalenhoef PA, Diederiks JP, Knottnerus JA, *et al*. A risk model for the prediction of recurrent falls in community-dwelling elderly: a prospective cohort study. *J Clin Epidemiol*. 2002 Nov;55(11):1088-94.

[3] Tinetti ME, Speechley M, Ginter SF. Risk factors for falls among elderly persons living in the community. *New England Journal of Medicine*. 1988;319:1701-1707.

[4] Downton JH, Andrews K, Prevalence, characteristics and factors associated with falls among the elderly living at home. *Aging*. 1991;3(3):219-28.

[5] Tinetti ME. Factors associated with serious injury during falls by ambulatory nursing home residents. *Journal of the American Geriatrics Society*. 1987;35:644-648.

[6] Nederlandse Vereniging voor Klinische Geriatrie (NVKG) Richtlijn Preventie van valincidenten bij ouderen (Guideline prevention of fall incidents in elderly patients, from the Dutch association of geriatrics), 2017. Available from: https://www.richtlijnendatabase.nl/richtlijn/preventie_van_valincidenten_bij_ouderen/startpagina_-_preventie_van_valincidenten.html, Feb 2018

[7] Helgadottir B, Laflamme L, Monarrez-Espino J, *et al*. Medication and fall injury in the elderly population: do individual demographics, health status and lifestyle matter? *BMC Geriatric* 2014;14:92

[8] Ham AC, Swart KM, Enneman AW., *et al*. Medication-related fall incidents in an older, ambulant population: I B-PROOF study. *Drugs Aging* 2014;31(12):917-27.

[9] Leipzig RM, Cumming RG, Tinetti ME. Drugs and falls in older people: a systematic review and meta-analysis: I. Psychotropic drugs. *J. Am. Geriatr. Soc*. 1999;47(1):30-9

[10] Leipzig RM, Cumming RG, Tinetti ME. Drugs and falls in older people: a systematic review and meta-analysis: II. Cardiac and analgesic drugs. *J. Am. Geriatr. Soc.* 1999; 47(1):40-50

[11] Woolcott JC, Richardson KJ, Wiens MO, *et al.* Meta-analysis of the impact of 9 medication classes on falls in elderly persons. *Arch Intern Med*. 2009;23;169(21):1952-60

[12] Janus SIM, Reinders GH, van Manen JG, *et al.* Psychotropic drug-related fall incidents in nursing home residents living in the eastern part of the Netherlands. *Drugs R D* (2017) 17:321-328

For peer review only

For peer review only - http://bmjopen.bmj.com/site/about/guidelines.xhtml

[13] Seppala LJ, Wermelink AMAT, de Vries M, *et al*. Fall-risk-increasing drugs: a systematic review and meta-analysis: II. Psychotropics. *J Am Med Dir Assoc.* 2018 Apr;19(4):371.e11-371.e17.

[14] Seppala LJ, van de Glind EMM, Daams JG, *et al.* Fall-risk-increasing drugs: a systematic review and meta-analysis: III. Others. *J Am Med Dir Assoc.* 2018 Apr;19(4):372.e1-372.e8.

[15] de Vries M, Seppala LJ, Daams JG, *et al.* Fall-risk-increasing drugs: a systematic review and meta-analysis: I. Cardiovascular drugs. *J Am Med Dir Assoc.* 2018 Apr;19(4):371.e1-371.e9.

[16] Bor A, Matuz M, Csatordai M, *et al.* Medication use and risk of falls among nursing home residents: a retrospective cohort study. *Int J Clin Pharm* (2017) 39:408-415

[17] Marcum ZA, Perera S, Thorpe JM, *et al.* Antidepressant use and recurrent falls in community-dwelling older adults: Findings from the health ABC study. *Ann Pharmacother.* 2016 July; 50(7):525-533

[18] Berry SD, Placide SG, Mostofsky E, *et al.* Antispychotic and benzodiazepine drug changes affect acute falls risk differently in the nursing home. *Journals of Gerontology: Med sciences,* 2016, Vol. 71, No. 2.

[19] Kuschel BM, Laflamme L, Möller J, The risk of fall injury in relation to commonly prescribed medications among older people-a Swedisch case-control study. *European Journal of Public Health*, Vol. 25, No. 3, 527-532.

[20] Maximos M, Chang F, Patel T, Risk of falls associated with antiepileptic drug use in ambulatory elderly populations: a systematic review. *Can Pharm J* (Ott) 2017; 150:101-111

[21] Matarese M, Ivziku D, Bartolozzi F, *et al.* Systematic review of fall risk screening tools for older patients in acute hospitals. *J Adv Nurs.* 2015 Jun; 71(6):1198-209

[22] Nunan S, Brown WC, Henwood T, *et al.* Fall risk assessment tools for use among older adults in long-term care settings: A systematic review of the literature. *Australas J Ageing.* 2018 Mar;37(1):23-33.

[23] Yazdani C, Hall S, Evaluation of the Medication fall risk score. *Am J Health-Syst Pharm.* 2017; 74:e32-9.

[24] Maurer MS, Burcham J, Cheng H, Diabetes Mellitus Is Associated With an Increased Risk of Falls in Elderly Residents of a Long-Term Care Facility. *Journal of Gerontology: MEDICAL SCIENCES*, 2005, Vol. 60A, No. 9, 1157–1162.

[25] Duh MS, Mody SH, Lefebvre P, *et al.*, Anaemia and the Risk of Injurious Falls in a Community-Dwelling Elderly Population. *Drugs Aging* 2008; 25 (4): 325-334

For peer review only

For peer review only - http://bmjopen.bmj.com/site/about/guidelines.xhtml

[26] Ambrosea AF, Geet P, Hausdorff JM, Risk factors for falls among older adults: A review of the literature. *Maturitas* 75 (2013) 51– 61.

[27] Dharmarajan TS, Avula S, Norkus EP, Anemia Increases Risk for Falls in Hospitalized Older Adults: An Evaluation of Falls in 362 Hospitalized, Ambulatory, Long-Term Care, and Community Patients. *Jamda* 2006.12.015

[28] Polinder-Bos HA, Emmelot-Vonk MH, Gansevoort RT, *et al.* High fall incidence and fracture rate in elderly dialysis patients. *The Netherlands Journal of Medicine*. Dec. 2014, Vol.72, No. 10

[29] Fehlberg EA, Lucero RJ, Weaver MT, Associations between hyponatraemia, volume depletion and the risk of falls in US hospitalized patients: a case–control study. *BMJ Open* 2017;7

[30] Jill Jin, Prevention of Falls in Older Adults, *JAMA*. 2018;319(16):1734

[31] Park H, Satoh H, Miki A, *et al.* Medications associated with falls in older people: systematic review of publications from a recent 5-year period. *Eur J Clin Pharmacol*. 2015 Dec;71(12):1429-40.

For peer review only

For peer review only - http://bmjopen.bmj.com/site/about/guidelines.xhtml

Figure 1 caption:

Figure 1: Inclusion of nursing home patients Zuyderland Medical Centre in part I of the study
* Model I is at the reference date (= day of the fall incident), model II is 3 days prior to the reference date and model III is 5 days prior to the reference date.

Figure 2 caption:

Figure 2: ROC curve fall risk predictive clinical rule

For peer review only

For peer review only - http://bmjopen.bmj.com/site/about/guidelines.xhtml

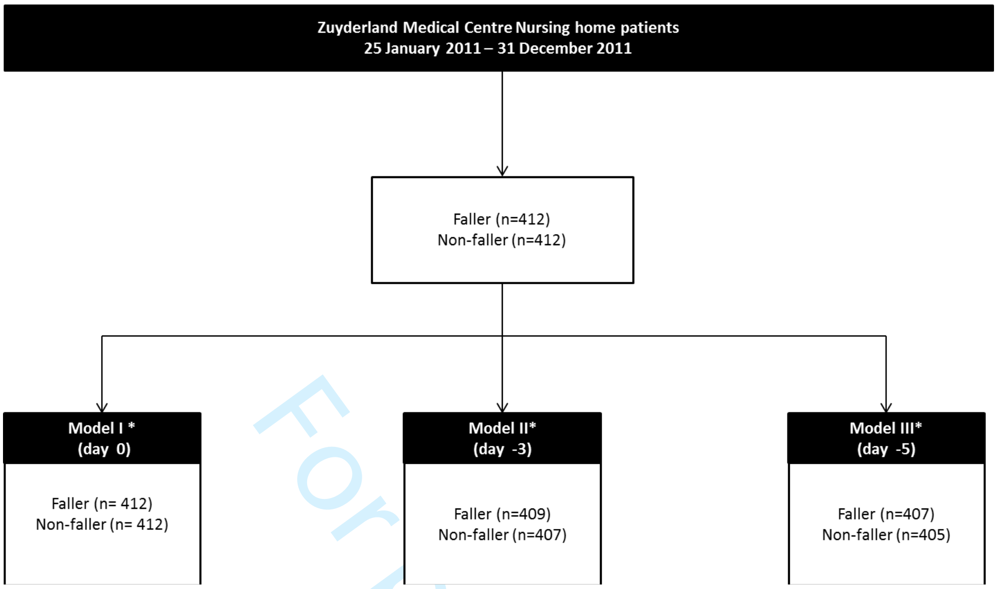

For peer review only

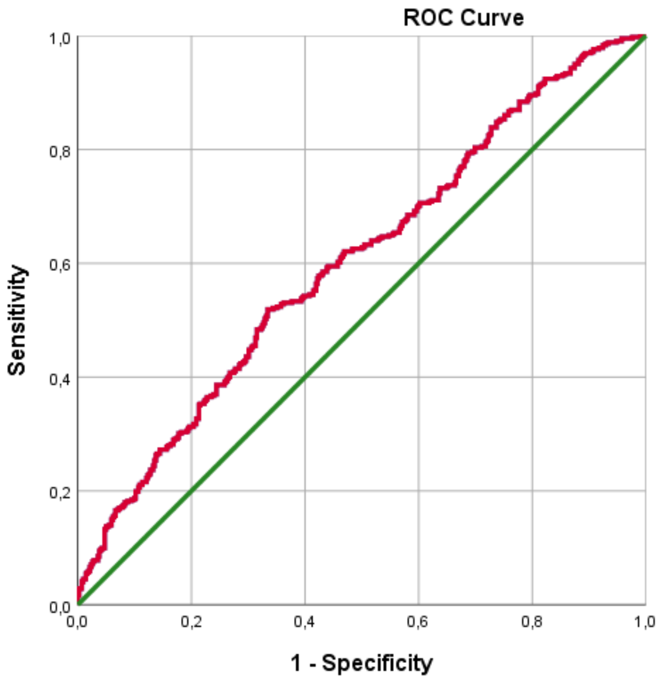

review only

For peer review only - http://bmjopen.bmj.com/site/about/guidelines.xhtml

Supplementary Table 1a: Potential fall risk factors, Model I (day 0)

Risk factors	Mean (SD)	Frequency (number)	% of total	OR	95% C.I. OR		p-value
					Upper	Lower	
**Age (y)**	82.43 (8.46)	-	-	1.044	1.026	1.063	**.000** *
**Age(y) centred**	0.03 (8.46)	-	-	1.035	1.015	1.055	**.001** *
**Age(y) centred^2**	71.57 (147.93)	-	-	.998	.996	.999	**.008** *
**Gender**	-	252 *M*, 572 *F*	30.58 *M*, 69.42 *F*	.561	.415	.758	**.000** *
**ATC unique**	8.49 (3.91)	-	-	1.035	.999	1.072	.059
**ATC unique≥5**		697	84.59	1.183	.810	1.729	.385
**ATC unique 0-5**		127	15.41	.845	.578	1.235	.385
**ATC unique 5- 10**		399	48.42	.864	.657	1.136	.296
**ATC unique 10-15**		226	27.43	1.103	.811	1.499	.531
**ATC unique ≥15**		72	8.74	1.640	1.000	2.690	**.050** *
**N06D**		97	11.77	6.057	3.474	10.558	**.000** *
**C01**		160	19.42	1.168	.827	1.651	.379
**N04A**		1	.12	*NA*	*NA*	*NA*	*NA*
**N06A**		253	30.70	1.394	1.035	1.878	**.029** *
**N03A**		75	9.10	.499	.304	.820	**.006** *
**R06A**		32	3.88	1.139	.561	2.313	.719
**N05A**		304	36.89	2.144	1.605	2.863	**.000** *
**C07**		199	24.15	.864	.628	1.190	.370
**S01ED**		24	2.91	1.414	0.621	3.222	0.409
**N05C**		280	33.98	1.417	1.060	1.894	**.019** *
**G04**		104	12.62	1.635	1.074	2.489	**.022** *
**C08**		80	9.71	.895	.564	1.420	.638
**C03**		281	34.10	1.177	.881	1.571	.270
**M03**		7	.85	.000	.000	*NA*	0.999
**C04AC**		0	.00	*NA*	*NA*	*NA*	*NA*
**C01C**		2	.24	1.000	.062	16.055	1.000
**N02A**		101	12.26	.589	.385	.903	**.015** *
**A02B**		318	38.59	.866	.654	1.147	.316
**C09**		211	25.61	1.039	.760	1.421	.811
**N07C**		20	2.43	1.000	.411	2.435	1.000
**N02B**		395	47.94	1.030	.783	1.355	.834
**N07AA**		3	.36	.498	.045	5.524	.571
**A10**		146	17.72	1.106	.772	1.582	.583
**C02**		6	.73	1.000	.200	4.988	1.000
**M01A**		54	6.55	1.083	.623	1.882	.778
**N07C**		20	2.43	1.000	.411	2.435	1.000
**N05B**		231	28.03	1.352	.996	1.835	**.053** *
**C10**		176	21.36	1.060	.759	1.479	.734
**R01BA**		0	.00	*NA*	*NA*	*NA*	*NA*
**C01A**		69	8.37	1.100	.671	1.804	.705
**C01B**		8	.97	1.000	.248	4.030	1.000
**C01D**		98	11.89	1.149	.753	1.754	.519
**N04**		37	4.49	.945	.488	1.828	.866
**N06DA**		41	4.98	5.217	2.284	11.917	**.000** *
**G04CA**		75	9.10	1.768	1.083	2.885	**.023** *

* statistically significant (p-value < 0.05)

NA = not available; SD = standard deviation; OR = Odds Ratio; C.I.= confidence interval; *M* = male, *F* = female

For peer review only - http://bmjopen.bmj.com/site/about/guidelines.xhtml

Supplementary Table 1b: Potential fall risk factors, Model II (day -3)

Risk factors	Mean (SD)	Frequency (number)	% of total	OR	95% C.I. OR		p-value
					Upper	Lower	
**Age (y)**	82.47 (8.39)	-	-	1.043	1.025	1.062	**.000** *
**Age(y) centred**	0.00 (8.39)	-	-	1.033	1.014	1.054	**.001** *
**Age(y) centred^2**	70.35 (143.45)	-	-	.998	.996	.999	**.007** *
**Gender**	-	246 *M*, 570 *F*	30.15% *M*, 69.85% *F*	.546	.403	.740	**.000** *
**ATC unique**	8.46 (3.90)	-	-	1.029	.993	1.066	.115
**ATC unique≥5**		690	84.56	1.044	.714	1.527	.824
**ATC unique 0-5**		126	15.44	.958	.655	1.401	.824
**ATC unique 5- 10**		401	49.14	.872	.662	1.147	.328
**ATC unique 10-15**		221	27.08	1.057	.776	1.439	.726
**ATC unique ≥15**		68	8.33	1.467	.887	2.428	.136
**N06D**		97	11.89	6.553	3.707	11.584	**.000** *
**C01**		161	19.73	1.216	.861	1.718	.268
**N04A**		1	.12	*NA*	*NA*	*NA*	*NA*
**N06A**		253	31.00	1.386	1.028	1.868	**.032** *
**N03A**		75	9.19	.529	.323	.866	**.011** *
**R06A**		32	3.92	1.133	.558	2.301	.729
**N05A**		304	37.25	2.044	1.530	2.730	**.000** *
**C07**		199	24.39	.858	.623	1.182	.350
**S01ED**		24	2.94	1.408	.618	3.208	.416
**N05C**		277	33.95	1.365	1.020	1.828	**.036** *
**G04**		104	12.75	1.554	1.022	2.362	**.039** *
**C08**		81	9.93	.916	.579	1.450	.708
**C03**		278	34.07	1.132	.847	1.512	.404
**M03**		7	.86	.000	.000	*NA*	.999
**C04AC**		0	.00	*NA*	*NA*	*NA*	*NA*
**C01C**		2	.25	.994	.062	15.954	.996
**N02A**		95	11.64	.568	.366	.883	**.012** *
**A02B**		314	38.48	.840	.634	1.115	.228
**C09**		211	25.86	1.059	.774	1.449	.720
**N07C**		19	2.33	1.107	.444	2.764	.827
**N02B**		384	47.06	.933	.709	1.229	.624
**N07AA**		3	.37	.495	.045	5.489	.567
**A10**		143	17.52	1.119	.779	1.608	.541
**C02**		6	.74	.994	.199	4.958	.994
**M01A**		53	6.50	.955	.547	1.667	.871
**N07C**		19	2.33	1.107	.444	2.764	.827
**N05B**		229	28.06	1.380	1.015	1.877	**.040** *
**C10**		179	21.94	1.037	.745	1.446	.828
**R01BA**		0	.00	*NA*	*NA*	*NA*	*NA*
**C01A**		68	8.33	1.133	.688	1.865	.625
**C01B**		8	.98	.994	.247	4.006	.993
**C01D**		100	12.25	1.194	.784	1.816	.409
**N04**		37	4.53	.940	.486	1.818	.853
**N06DA**		40	4.90	6.071	2.519	14.628	**.000** *
**G04CA**		75	9.19	1.653	1.015	2.689	**.043** *

* statistically significant (p-value < 0.05)

NA = not available; SD = standard deviation; OR = Odds Ratio; C.I.= confidence interval; *M* = male, *F* = female

For peer review only

For peer review only - http://bmjopen.bmj.com/site/about/guidelines.xhtml

Supplementary Table 1c: Potential fall risk factors, Model III (day -5)

Risk factors	Mean (SD)	Frequency (number)	% of total	OR	95% C.I. OR		p-value
					Upper	Lower	
**Age (y)**	82.52 (8.36)	-	-	1.042	1.024	1.061	**.000** *
**Age(y) centred**	0.00 (8.36)	-	-	1.033	1.013	1.053	**.001** *
**Age(y) centred^2**	69.75 (143.05)	-	-	.998	.996	.999	**.010** *
**Gender**	-	243 *M*, 569 *F*	29.93 *M*, 70.07 *F*	.536	.395	.728	**.000** *
**ATC unique**	8.44 (3.93)	-	-	1.023	.987	1.059	.214
**ATC unique≥5**		684	84.24	1.083	.742	1.581	.679
**ATC unique 0-5**		128	15.76	.923	.633	1.348	.679
**ATC unique 5- 10**		395	48.65	.906	.688	1.194	.484
**ATC unique 10-15**		223	27.46	1.083	.796	1.474	.613
**ATC unique ≥15**		66	8.13	1.296	.781	2.152	.316
**N06D**		94	11.58	6.833	3.801	12.285	**.000** *
**C01**		160	19.70	1.198	.847	1.695	.306
**N04A**		1	.12	*NA*	*NA*	*NA*	*NA*
**N06A**		251	30.91	1.357	1.006	1.830	**.045** *
**N03A**		74	9.11	.541	.330	.888	**.015** *
**R06A**		30	3.69	.995	.480	2.063	.989
**N05A**		298	36.70	1.973	1.475	2.639	**.000** *
**C07**		196	24.14	.868	.629	1.198	.391
**S01ED**		24	2.96	1.408	.618	3.208	.416
**N05C**		273	33.62	1.400	1.045	1.878	**.024** *
**G04**		103	12.68	1.457	.958	2.215	.078
**C08**		78	9.61	.940	.589	1.499	.794
**C03**		277	34.11	1.120	.837	1.498	.446
**M03**		7	.86	.000	.000	*NA*	.999
**C04AC**		0	.00	*NA*	*NA*	*NA*	*NA*
**C01C**		2	.25	.994	.062	15.955	.996
**N02A**		91	11.21	.586	.374	.916	**.019** *
**A02B**		312	38.42	.840	.633	1.114	.226
**C09**		209	25.74	1.060	.773	1.451	.719
**N07C**		19	2.34	1.107	.444	2.764	.827
**N02B**		383	47.17	.905	.687	1.194	.481
**N07AA**		3	.37	.495	.045	5.489	.567
**A10**		140	17.24	1.065	.739	1.534	.736
**C02**		6	.74	.994	.199	4.959	.994
**M01A**		54	6.65	.918	.528	1.597	.761
**N07C**		19	2.34	1.107	.444	2.764	.827
**N05B**		228	28.08	1.332	.980	1.812	.067
**C10**		175	21.55	1.038	.743	1.451	.826
**R01BA**		0	.00	*NA*	*NA*	*NA*	*NA*
**C01A**		68	8.37	1.133	.688	1.865	.625
**C01B**		8	.99	.994	.247	4.006	.993
**C01D**		99	12.19	1.168	.766	1.781	.469
**N04**		37	4.56	.940	.486	1.818	.853
**N06DA**		39	4.80	7.307	2.827	18.885	**.000** *
**G04CA**		75	9.24	1.555	.958	2.524	.074

* statistically significant (p-value < 0.05)

NA = not available; SD = standard deviation; OR = Odds Ratio; C.I.= confidence interval; *M* = male, *F* = female

TRIPOD Checklist: Prediction Model Development

Section/Topic	Item	Checklist Item	Page
**Title and abstract**			
Title	1	Identify the study as developing and/or validating a multivariable prediction model, the target population, and the outcome to be predicted.	1
Abstract	2	Provide a summary of objectives, study design, setting, participants, sample size, predictors, outcome, statistical analysis, results, and conclusions.	3
**Introduction**			
Background and objectives	3a	Explain the medical context (including whether diagnostic or prognostic) and rationale for developing or validating the multivariable prediction model, including references to existing models.	5-6
	3b	Specify the objectives, including whether the study describes the development or validation of the model or both.	6
**Methods**			
Source of data	4a	Describe the study design or source of data (e.g., randomized trial, cohort, or registry data), separately for the development and validation data sets, if applicable.	7
	4b	Specify the key study dates, including start of accrual; end of accrual; and, if applicable, end of follow-up.	7
Participants	5a	Specify key elements of the study setting (e.g., primary care, secondary care, general population) including number and location of centres.	7
	5b	Describe eligibility criteria for participants.	7
	5c	Give details of treatments received, if relevant.	-
Outcome	6a	Clearly define the outcome that is predicted by the prediction model, including how and when assessed.	7
	6b	Report any actions to blind assessment of the outcome to be predicted.	-
Predictors	7a	Clearly define all predictors used in developing or validating the multivariable prediction model, including how and when they were measured.	7-8
	7b	Report any actions to blind assessment of predictors for the outcome and other predictors.	-
Sample size	8	Explain how the study size was arrived at.	8-9
Missing data	9	Describe how missing data were handled (e.g., complete-case analysis, single imputation, multiple imputation) with details of any imputation method.	13-16
Statistical analysis methods	10a	Describe how predictors were handled in the analyses.	7-9
	10b	Specify type of model, all model-building procedures (including any predictor selection), and method for internal validation.	7-9
	10d	Specify all measures used to assess model performance and, if relevant, to compare multiple models.	7-9
Risk groups	11	Provide details on how risk groups were created, if done.	-
**Results**			
Participants	13a	Describe the flow of participants through the study, including the number of participants with and without the outcome and, if applicable, a summary of the follow-up time. A diagram may be helpful.	figure 1
	13b	Describe the characteristics of the participants (basic demographics, clinical features, available predictors), including the number of participants with missing data for predictors and outcome.	table 1
Model development	14a	Specify the number of participants and outcome events in each analysis.	suplementary tables
	14b	If done, report the unadjusted association between each candidate predictor and outcome.	suplementary tables
Model specification	15a	Present the full prediction model to allow predictions for individuals (i.e., all regression coefficients, and model intercept or baseline survival at a given time point).	table 3
	15b	Explain how to the use the prediction model.	table 3
Model performance	16	Report performance measures (with CIs) for the prediction model.	table 4
**Discussion**			
Limitations	18	Discuss any limitations of the study (such as nonrepresentative sample, few events per predictor, missing data).	13-16
Interpretation	19b	Give an overall interpretation of the results, considering objectives, limitations, and results from similar studies, and other relevant evidence.	13-16
Implications	20	Discuss the potential clinical use of the model and implications for future research.	13-16
**Other information**			
Supplementary information	21	Provide information about the availability of supplementary resources, such as study protocol, Web calculator, and data sets.	-
Funding	22	Give the source of funding and the role of the funders for the present study.	4

We recommend using the TRIPOD Checklist in conjunction with the TRIPOD Explanation and Elaboration document.

BMJ Open

Fall Incidents in Nursing home residents: DEvelopment of a predictive clinical Rule (FINDER)

Journal:	*BMJ Open*
Manuscript ID	bmjopen-2020-042941.R2
Article Type:	Original research
Date Submitted by the Author:	15-Feb-2021
Complete List of Authors:	Milosevic, Vanja; Zuyderland Medical Centre Sittard-Geleen, Clinical Pharmacy, Pharmacology and Toxicology; Elkerliek Ziekenhuis, Clinical Pharmacy<br>Linkens, Aimee; Maastricht University Medical Centre+, Internal Medicine; Erasmus MC, University Medical Center Rotterdam<br>Winkens, Bjorn; Maastricht University, Methodology and Statistics<br>Hurkens, Kim; Zuyderland Medisch Centrum Heerlen, Geriatric Medicine, Department of Internal Medicine<br>Wong, Dennis; Zuyderland Medical Centre Sittard-Geleen, Clinical Pharmacy, Pharmacology and Toxicology<br>van Oijen, Brigit; Zuyderland, Clinical Pharmacy, Pharmacology and Toxicology<br>van der Kuy, Hugo; Erasmus MC, Clinical Pharmacy<br>Mestres-Gonzalvo, Carlota; Maastricht University Medical Centre+, Clinical Pharmacy and Toxicology
**Primary Subject Heading**:	Pharmacology and therapeutics
Secondary Subject Heading:	Geriatric medicine, Medical management
Keywords:	CLINICAL PHARMACOLOGY, GENERAL MEDICINE (see Internal Medicine), GERIATRIC MEDICINE

SCHOLARONE™
Manuscripts

BMJ

I, the Submitting Author has the right to grant and does grant on behalf of all authors of the Work (as defined in the below author licence), an exclusive licence and/or a non-exclusive licence for contributions from authors who are: i) UK Crown employees; ii) where BMJ has agreed a CC-BY licence shall apply, and/or iii) in accordance with the terms applicable for US Federal Government officers or employees acting as part of their official duties; on a worldwide, perpetual, irrevocable, royalty-free basis to BMJ Publishing Group Ltd ("BMJ") its licensees and where the relevant Journal is co-owned by BMJ to the co-owners of the Journal, to publish the Work in this journal and any other BMJ products and to exploit all rights, as set out in our licence.

The Submitting Author accepts and understands that any supply made under these terms is made by BMJ to the Submitting Author unless you are acting as an employee on behalf of your employer or a postgraduate student of an affiliated institution which is paying any applicable article publishing charge ("APC") for Open Access articles. Where the Submitting Author wishes to make the Work available on an Open Access basis (and intends to pay the relevant APC), the terms of reuse of such Open Access shall be governed by a Creative Commons licence – details of these licences and which Creative Commons licence will apply to this Work are set out in our licence referred to above.

Other than as permitted in any relevant BMJ Author's Self Archiving Policies, I confirm this Work has not been accepted for publication elsewhere, is not being considered for publication elsewhere and does not duplicate material already published. I confirm all authors consent to publication of this Work and authorise the granting of this licence.

For peer review only

Title

Fall Incidents in Nursing home residents: DEvelopment of a predictive clinical Rule (FINDER)

Authors

Vanja Milosevic[1,2,6]

vmilosevic@elkerliek.nl

Aimee Linkens[3,6]

aimee.linkens@mumc.nl

Bjorn Winkens[4]

bjorn.winkens@maastrichtuniversity.nl

Kim Hurkens[5]

k.hurkens@zuyderland.nl

Dennis Wong[1]

d.wong@zuyderland.nl

Brigit van Oijen[1]

br.vanoijen@zuyderland.nl

Hugo van der Kuy[6]

h.vanderkuy@erasmusmc.nl

Carlota Mestres-Gonzalvo[7]

c.mestres@mumc.nl

[1]Department of Clinical Pharmacy, Pharmacology and Toxicology, Zuyderland Medical Centre, Dr. H van der Hoffplein 1, 6162 BG, Sittard-Geleen and Henri Dunantstraat 5, 6419 PC Heerlen, The Netherlands.

[2]Department of Clinical Pharmacy, Elkerliek Hospital, Wesselmanlaan 25, 5707 HA Helmond, The Netherlands.

[3]Department of Internal Medicine, Maastricht University Medical Centre+, P. Debyelaan 25, 6229 HX Maastricht, The Netherlands.

[4]Department of Methodology and Statistics, Faculty of Health, Medicine and Life Sciences, Maastricht University, P. Debyeplein 1, 6229 HA Maastricht, The Netherlands.

[5]Department of Internal Medicine sect. of Geriatric Medicine, Zuyderland Medical Centre, Dr. H van der Hoffplein 1, 6162 BG, Sittard-Geleen and Henri Dunantstraat 5, 6419 PC Heerlen, The Netherlands.

For peer review only

[6]Erasmus MC, University Medical Center Rotterdam, Department of Hospital Pharmacy, Postbus 2040, 3000 CA Rotterdam, The Netherlands.

[7]Department of Clinical Pharmacy and Toxicology, Maastricht University Medical Centre+, P. Debyelaan 25, 6229 HX Maastricht, The Netherlands.

Corresponding Author:

Prof. dr. Hugo van der Kuy

Department of Clinical Pharmacy

Erasmus Medical Centre

Postbus 2040

3000 CA Rotterdam

The Netherlands

Email: h.vanderkuy@erasmusmc.nl

Keywords

Fall risk, Decision Support System, nursing home residents, ROC Curve, Logistic Models.

Word count: 3979 (without abstract, strength and limitations, literature, figures and tables)

Number of tables: 3

Number of figures: 2

Number of supplementary tables: 3 (1a,b and c)

Number of supplementary texts: 0

For peer review only

For peer review only - http://bmjopen.bmj.com/site/about/guidelines.xhtml

ABSTRACT (298)

Objectives: To develop (part I) and validate (part II) an electronic fall risk clinical rule (CR) to identify nursing home residents (NH-residents) at risk for a fall incident.

Design: Observational, retrospective case-control study.

Setting: Nursing homes

Participants: A total of 1668 (824 in part I, 844 in part II) NH-residents from The Netherlands were included. Data of participants from part I were excluded in part II.

Primary and secondary outcome measures: Development and validation of a fall risk CR in NH-residents. Logistic regression analysis was conducted to identify the fall risk-variables in part I. With these, three CRs were developed (i.e. at the day of the fall incident and three days and five days prior to the fall incident). The overall prediction quality of the CRs were assessed using the Area Under the Receiver Operating Characteristics (AUROC), and a cut-off value was determined for the predicted risk ensuring a sensitivity ≥ 0.85. Finally, one CR was chosen and validated in part II using a new retrospective dataset.

Results: Eleven fall risk-variables were identified in part I. The AUROCs of the three CRs form part I were similar: the AUROC for model I, II and III were 0.714 (95% CI 0.679 to 0.748), 0.715 (95%CI 0.680 to 0.750) and 0.709 (95%CI 0.674 to 0.744), respectively. Model III (i.e. 5 days prior to the fall incident) was chosen for validation in part II. The validated AUROC of the CR, obtained in part II, was 0.603 (95%CI 0.565 to 0.641) with a sensitivity of 83.41% (95% CI 79.44% to 86.76%) and a specificity of 27.25%(95% CI 23.11% to 31.81%).

Conclusion: Medication data and resident characteristics alone are not sufficient enough to develop a successful CR with a high sensitivity and specificity to predict fall risk in NH-residents.

Trial registration: not available

STRENGHTS AND LIMITATIONS OF THIS STUDY

- Currently there is no golden standard in the Netherlands regarding the use of a specific tool in the prevention of fall risk in NH-residents.
- This study was based on electronic data and variables that are currently available in daily practice and therefore easy to implement. However, fall risk factors that were not available electronically are lacking.

For peer review only

For peer review only - http://bmjopen.bmj.com/site/about/guidelines.xhtml

- The CR was developed and validated using two different retrospective data sets of the same nursing homes and therefore lacks a prospective validation in a different nursing home setting.

FUNDING

This research received no specific grant from any funding agency in the public, commercial or not-for-profit sectors.

Competing Interests

All authors have declared that no competing interests exist.

Abbreviations

Clinical rule (CR), nursing home residents (NH-residents), Clinical Decision Support System (CDSS), fall risk increasing drugs (FRIDs), receiver operating characteristics (ROC), area under the receiver operating characteristics (AUROC),

For peer review only

For peer review only - http://bmjopen.bmj.com/site/about/guidelines.xhtml

INTRODUCTION / BACKGROUND (756)

Worldwide there are 646,000 falls every year which result in death, making falls the second leading cause of unintended death [1]. Falls are common in elderly people aged 65 years and over. According to the World Health Association, 28-35% of the elderly fall each year [1]. This percentage increases with age (e.g. 32-42% in the age group of 70 years and older) as does the fatal falls rate [2-4]. However, not only age seems to be related to the risk of falls, the frailty level is also a risk factor. For example, elderly who live in nursing homes have higher falling rates compared to those living in the community [5]. The incidence of falls in NH-residents ranges from 30 to 50% per year [1,6].
The ageing population challenges us with the question: how to prevent falls?

Falls occur as a result of a variety of circumstances, more specifically, there are four different dimensions involved in fall incidents: biological (e.g. race, gender, age, (multi)-morbidity), environmental (e.g. building design, insufficient light), socio-economic (e.g. inadequate housing, limited access to social services and health), and behavioural factors (e.g. polypharmacy, inappropriate foot ware) [1].

Focusing on behavioural aspects, specifically on medication, several studies have been conducted to investigate the effect of medication on fall risk [1,7-20]. These studies have shown that the risk of falls resulting in injuries and/or death, increases with increasing numbers of drugs used in the elderly [7]. Other studies investigated the association of different drug classes with an increased risk of falls [11-20]. Examples of identified drug classes with an increased fall risk are anti-arrhythmic drugs, antidepressants, psychotropic drugs, and diuretics [1,8-11].

Different strategies and tools to prevent fall incidents are developed [21]. Although a healthcare providers' clinical judgement is one of the possible strategies, a tool based on outcome measures would provide for a more objective and formal risk factor identification. However, given the multifactorial aspect and complexity of variables of fall incidents, it is extremely difficult to obtain a tool with a high sensitivity and specificity, and one that is easy to implement in practice [1,21,22].

The current tools differ in number and type of fall risk factors included (e.g. multifactorial assessment tools, functional mobility or medication related assessment tools). Also, the applicability is often restricted to one specific setting or subpopulation (e.g. hospital, nursing

For peer review only - http://bmjopen.bmj.com/site/about/guidelines.xhtml

For peer review only

home, ambulatory). In The Netherlands, there is still no recommendation for one specific tool in the prevention of fall risk in NH-residents, leading to local differences regarding fall risk management.

Of the most commonly used tools for the prediction of fall risk (i.e. Hendrich Fall Risk Model I and II (HFRM I and II), St. Thomas's risk assessment tool in falling elderly inpatients (STRATIFY) and the Morse Fall Scale (MFS)), only the HFRM II tool makes use of information regarding the use of anti-epileptics and benzodiazepines. Both MFS and STRATIFY have no medication related fall risk factors included in the tool [6,23].

Nunan et al. [22], conducted a systematic review regarding fall risk assessment tools in the long-term care setting. In general, the following remarks can be made on current tools for prevention of fall incidents in the long-term care setting: they are either not validated, or have a low sensitivity and/or specificity (lower than 70%), and/or the practical applicability is below the desired level [22]. Therefore, there is still room for improvement.

Given the multifactorial aspect of fall incidents, a tool that combines all of the above-described dimensions (i.e. biological, environmental, socio-economic and behavioural) involved in fall incidents, would probably give the most accurate results. Currently there is no such tool available.

Our goal is to focus on all of the electronically available data of interest in fall incidents, aiming to combine as many different dimensions involved in fall incidents as possible. Thereby we will include age, gender, the presence of specific medication (expressed as ATC-codes i.e. codes from the Anatomical Therapeutic Chemical classification system), the number of unique ATC-codes and laboratory parameters. The specific ATC-codes and laboratory parameters that will be included in this study, are those from which there is evidence from other studies that there is a (possible) association with the fall risk.

With a Clinical Decision Support System (CDSS), one is able to combine different data, such as resident characteristics, laboratory and medication data, with the goal to generate specific alerts per resident. Developing and implementing a predictive CR for fall risk in an electronic CDSS could therefore be helpful for healthcare professionals in order to prevent falls in those who are at increased risk.

For peer review only

For peer review only - http://bmjopen.bmj.com/site/about/guidelines.xhtml

Objective (35)

The objective of this study is to develop (part I) and validate (part II) a fall risk CR that can be used in an electronic CDSS to identify NH-residents at risk for a fall incident.

METHODS (1091)

Design (61)

An observational retrospective case-control study was conducted in two parts. In part I, a predictive fall risk CR for implementation in an electronic CDSS was developed in a dataset of NH-residents. In part II, the developed CR was retrospectively validated in a different dataset of NH-residents.

For both parts of the study, data from NH-residents from Sittard-Geleen, The Netherlands, were used.

Participants selection (42)

Residents of nursing homes were selected. The population consists of both somatic and non-somatic residents. For part I of the study, no exclusion criteria were used. In part II of the study, participants (fallers and non-fallers) from part I were excluded.

Outcome (163)

The outcome that is predicted by the fall risk CR is a fall.

In part I, NH-residents who have fallen at least once within the period of January 25th, 2011 until December 31th, 2011, were defined as *fallers (cases)*. NH-residents who have not fallen in the same period, were defined as *non-fallers (controls)*. In part II the same design was used as in part I. Part II was conducted in a new dataset of NH-residents in the period of January 25th, 2016 until December 31th, 2016.

In both parts of the study, the date of the first fall of the faller within the study period was set as the reference date. As a pragmatic approach, we chose to collect the data of the residents in the control group at similar dates (i.e. reference dates for controls) as those of the cases.

For peer review only

Therefore, each non-faller was linked at random with a faller. As a result all risk factors might differ between cases and controls.

Potential predictors (235)

Hospital and nursing home electronic systems were used to extract data on resident characteristics (i.e. age and gender), laboratory data, medication (number and type of medicines), and reports on fall incidents. More specifically, data on medication were collected at the reference date and 3 and 5 days prior to the reference date, while laboratory data of approximately up to 3 months prior to the reference date were retrieved.

The laboratory parameters intended for analysis, are laboratory parameters known from literature to be associated with fall risk: albumin, sodium, potassium, creatinine, kidney clearance (MDRD), glucose and hemoglobin [24-29]. Regarding the medication data, the residents medication list was screened for presence of 35 different ATC-codes all of which are shown to have a (possible) association with fall risk [6,8-20]. Also the number of unique ATC-codes was collected.

Finally, electronically available resident characteristics, such as age and gender at time of the reference date, as well as the reports on fall incidents (date of the report), were obtained.

The described above dataset for part I was analysed in order to develop a predictive fall risk CR for an electronic CDSS based on relevant risk factors for the prediction of the fall risk in NH-residents. In part II, the developed CR was validated by determining its specificity and sensitivity using a new dataset of Zuyderland MC NH-residents obtained in the period of January 25th, 2016 until December 31th, 2016.

Statistical analysis (342)

Statistical analyses were performed using IBM SPSS Statistics for Windows (version 21.0. Armonk, NY: IBM Corp.). Two-sided p-values smaller than or equal to 0.05 were considered statistically significant.

For part I of the study, logistic regression analysis was used to identify statistically significant risk factors for the prediction of the fall risk, where variables significantly related to the

For peer review only

For peer review only - http://bmjopen.bmj.com/site/about/guidelines.xhtml

outcome were included in the multiple logistic regression model. Three predictive models were created as medication data were collected at three different moments, i.e. at the time of the reference date (model I), 3 days prior to the reference date (model II), and 5 days prior to the reference date (model III). As laboratory data (collected up to approximately 3 months prior to the reference date) was only available in 411 out of 824 NH-residents (49.9%), the models were only based on medication data (specific ATC codes), age and gender. One model was eventually chosen for development and validation of the predictive CR.

Linearity assumption was checked for numerical variables by centering these variables and adding the quadratic centered term to the model. If this quadratic term was significant, the linear as well as quadratic term for this variable were included in the multiple regression model. The predictive CR (multiple linear regression model) was then used to obtain a predictive score which quantifies the estimated fall risk of the individual nursing home resident according to his/her specific risk factors. The overall predictive quality of the CR was determined by the c-statistic, i.e. the area under the receiver operating characteristics (AUROC) curve. Additionally, an optimal cut-off point for the estimated fall risk was obtained by two criteria: A) maximizing the Youden's J-statistic, i.e. sensitivity+specificity-1, and B) maximizing specificity where the sensitivity is at least 85%.

The predictive CR was applied to the dataset from part II of the study, where the optimal cut-off value obtained from part I was then used to determine the validated prognostic values of the CR. To obtain 95% confidence intervals around these prognostic values an online calculator (http://vassarstats.net/clin1.html) was used.

Sample size calculation (232)

The sample size was estimated for a CR that could consist of 30-40 risk factors. This number of risk factors was based on the number of different ATC-codes (fall risk increasing drugs (FRIDs), known from literature), use of polypharmacy (≥5 different ATC-codes in chronic use), the number of relevant laboratory data, and the resident characteristics age and gender [6,8-20,24-29]. The multiple logistic regression model requires at least 10 fallers for each individual risk factor. In order to obtain a representative incidence of fallers (about 50% per year) in the study population, also 10 non-fallers are needed per individual risk factor. This means that a total of approximately 800 NH-residents (at least 400 fallers and 400 non-

For peer review only

fallers) were needed to carry out part I of the study, and another 800 NH-residents were needed for part II of the study (at least 400 fallers and 400 non-fallers).

These numbers would be large enough to ensure precise estimates of sensitivity and specificity of the predictive CR. More specifically, the width of the 95% confidence interval (CI) of sensitivity and specificity of the predictive CR would be maximally equal to 2*1.96*0.5/√n = 1.96/√n, where n equals the number of fallers for sensitivity and number of non-fallers for specificity. Therefore, with at least 400 fallers and 400 non-fallers, the 95%CI of sensitivity and specificity of the predictive CR would not be wider than 0.10 (1.96/√400 = 0.098).

Patient and Public Involvement (20)

Patients or the public were not involved in the design, or conduct, or reporting, or dissemination plans of our research.

RESULTS (333)

Participants

A total of 824 NH-residents (412 fallers and 412 non-fallers) in the time period of January 25th, 2011 up to December 31th, 2011 were included in part I of the study (Figure 1). Characteristics of the included NH-residents for part I of the study are shown in Table 1.

Table 1: Resident characteristics in part I of the study

	**Model I (day 0)**			**Model II (day -3)**			**Model III (day -5)**		
	Faller (n=412)	Non-faller (n=412)	Total (n=824)	Faller (n=409)	Non-faller (n=407)	Total (n=816)	Faller (n=407)	Non-faller (n=405)	Total (n=812)
**Mean Age ± SD* (y)**	83.91 ±6,87	80.96 ±9,59	82.43 ±8,46	83.89 ±6,86	81.05 ±9,49	82.47 ±8,39	83.90 ±6,88	81.13 ±9,42	82.52 ±8,36
**Male**	151 (36.7%)	101 (24.5%)	252 (30.6%)	149 (36.4%)	97 (23.8%)	246 (30.1%)	148 (36.4%)	95 (23.5%)	243 (29.9%)
**Female**	261 (63.3%)	311 (75.5%)	572 (69.4%)	260 (63.6%)	310 (76.2%)	570 (69,9%)	259 (63.6%)	310 (76.5%)	569 (70.1%)

*SD= standard deviation; y =years

For peer review only

For peer review only - http://bmjopen.bmj.com/site/about/guidelines.xhtml

290 **Model development and specification**

291 Table 2 shows the variables that were selected from literature and included for analysis in
292 part I of the study.

293

For peer review only

For peer review only - http://bmjopen.bmj.com/site/about/guidelines.xhtml

Table 2: Fall risk variables

Variable	Description
**Polyfarmacy**	
ATCunique	Number of unique ATC codes
ATCunique ≥5	Presence of 5 or more unique ATC codes (yes=1, no=0)
ATCunique 0 - 5	Presence of 0-5 unique ATC codes (yes=1, no=0)
ATCunique 5 - 10	Presence of 5-10 unique ATC codes (yes=1, no=0)
ATCunique10 - 15	Presence of 10-15 unique ATC codes (yes=1, no=0)
ATCunique ≥ 15	Presence of 15 or more unique ATC codes (yes=1, no=0)
**Resident characteristics**	
Gender	Male / Female
Age	Age in years
Age_c	Age in years, centered
Age_c2	Age in years, centered quadratic term
**Presence of specific ATC codes (yes=1, no=0)**	
A02B	DRUGS FOR PEPTIC ULCER AND GASTRO-OESOPHAGEAL REFLUX DISEASE (GORD)
A10	DRUGS USED IN DIABETES
C01	CARDIAC THERAPY
C01A	CARDIAC GLYCOSIDES
C01B	ANTIARRHYTHMICS, CLASS I AND III
C01C	CARDIAC STIMULANTS EXCL. CARDIAC GLYCOSIDES
C01D	VASODILATORS USED IN CARDIAC DISEASES
C02	ANTIHYPERTENSIVES
C03	DIURETICS (cardiovascular system)
C04AC	Nicotinic acid and derivatives (peripheral vasodilators)
C07	BETA BLOCKING AGENTS (cardiovascular system)
C08	CALCIUM CHANNEL BLOCKERS (cardiovascular system)
C09	AGENTS ACTING ON THE RENIN-ANGIOTENSIN SYSTEM
C10	LIPID MODIFYING AGENTS
G04	UROLOGICALS
G04CA	Alpha-adrenoreceptor antagonists (urologicals)
M01A	ANTIINFLAMMATORY AND ANTIRHEUMATIC PRODUCTS, NON-STEROIDS
M03	MUSCLE RELAXANTS
N02A	OPIOIDS (analgetics)
N02B	OTHER ANALGESICS AND ANTIPYRETICS
N03A	ANTIEPILEPTICS
N04	ANTI-PARKINSON DRUGS
N04A	ANTICHOLINERGIC AGENTS (Anti-parkinson drugs)
N05A	ANTIPSYCHOTICS
N05B	ANXIOLYTICS
N05C	HYPNOTICS AND SEDATIVES
N06A	ANTIDEPRESSANTS
N06D	ANTI-DEMENTIA DRUGS
N06DA	Anticholinesterases (psychoanaleptics)
N07AA	Anticholinesterases (parasympathomimetics)
N07C	ANTIVERTIGO PREPARATIONS
R01BA	Sympathomimetics (respiratory system)
R06A	ANTIHISTAMINES FOR SYSTEMIC USE
S01ED	Beta blocking agents (ophthalmologicals)

For peer review only

For peer review only - http://bmjopen.bmj.com/site/about/guidelines.xhtml

From the 44 variables studied in total (Table 2), only 11 variables were found to be significantly related to fall risk (supplementary table 1a,b,c). The multiple logistic regression models consisting of these 11 variables, were applied to the data. The AUROC for model I, II and III were 0.714 (95% CI 0.679-0.748), 0.715 (95%CI 0.680-0.750) and 0.709 (95%CI 0.674-0.744), respectively.

For the development of the predictive CR, model III (5 days before reference day) was chosen, since the predictive quality of models I, II and III were similar. Based on the significant fall risk variables, the following predictive CR was developed to calculate the fall risk (Table 3).

Table 3: Fall risk predictive algorithm (*CR*) for model III (5 days prior to reference day).

Fall risk predictive CR (model III) in NH-residents:
Fall risk = 1/(1+Exp(-Linear predictor).

Linear predictor = 0.7799 -0.6802*V1 + 0.0460*V2- -0.0016*V3+ 1.6442*V4 + 0.2664*V5 -0.4763*V6 + 0.4267*V7 + 0.2368*V8 + 0.0001*V9 -0.3780*V10+0.2927*V11

Predictive fall risk factors:

V1	Gender (1 = male, 2 = female)
V2	Age_c = age -82,52 (age centered, linear term)
V3	Age_c2 = $(age\_c)^2$ (age centered, quadratic term)
V4	Presence of medicines with ATC code NO6D (yes=1, no=0)
V5	Presence of medicines with ATC code N06A (yes=1, no=0)
V6	Presence of medicines with ATC code N03A (yes=1, no=0)
V7	Presence of medicines with ATC code N05A (yes=1, no=0)
V8	Presence of medicines with ATC code N05C (yes=1, no=0)
V9	Presence of medicines with ATC code G04 (yes=1, no=0)
V10	Presence of medicines with ATC code N02A (yes=1, no=0)
V11	Presence of medicines with ATC code N05B (yes=1, no=0)

The optimal cut-off value for fall risk, using Youden's J-statistic, was 0.5590, resulting in a sensitivity of 46.9% and a specificity of 81.7%. If the optimal cut-off value was based on the second criterion, where the sensitivity should at least be 85.0%, then the associated cut-off value was 0.4009, resulting in a sensitivity of 85.0% and a specificity of 40.7%.

For peer review only

For peer review only - http://bmjopen.bmj.com/site/about/guidelines.xhtml

Model performance

In part II of the study, the developed CR was validated in a cohort of 844 NH-residents (422 fallers and 422 non-fallers) in the time period of January 25th, 2016 to December 31th, 2016. Applying the predictive CR on this cohort of NH-residents resulted in an AUROC of 0.603 (95%CI 0.565-0.641) (Figure 2). If the cut-off value for the fall risk was based on the second criterion (sensitivity ≥ 85%), then the validated sensitivity and specificity were 83.4% (95% CI 79.4% - 86.8%) and 27.3% (95% CI 23.1%-31.8%), respectively.

DISCUSSION AND CONCLUSION (1760)

A CR for NH-residents was developed and validated. This resulted in a CR which can be used to predict the fall risk 5 days prior to the fall incident, based on 11 variables, which were found to be significantly related to fall risk as shown in part I of the study. After identification of the 11 variables, three prediction models were compared: a fall risk prediction model at the reference date, 3 days prior to the reference date and 5 days prior to the reference date. Since the AUROC of all three models was similar, we choose to validate (in part II) the model to predict the fall incidence 5 days prior to the actual event. This decision was made because in daily practice, it is preferred to predict the fall incident as early as possible in order to take the necessary actions.

Since fall incidents are a massive problem in society and often result in a hospital admission or high mortality rates, we have chosen to set the sensitivity of the prediction model at 85.0%. A sensitivity of 85.0% results in missing less fall incidents.

A critical remark is that validation of the CR (part II of the study) resulted in 83.4% sensitivity and 27.3% specificity. A specificity of 27.3% means approximately 73% of the residents who would not fall within 5 days, would incorrectly be classified as having an increased fall risk. Since NH-residents are vulnerable, it may be useful to review the medication regardless of the fall risk. Also, the actions that are taken in fall risk prevention are mostly not drug related, but include e.g. exercise therapy, psychological health, vision, environmental conditions, diet and nutrition and sometimes may include medication [30]. In addition, it is still up to the healthcare professional to review per individual whether actions are needed to be taken to prevent falls and if so, which actions need to be taken, customized to the subject’s needs.

For peer review only

For peer review only - http://bmjopen.bmj.com/site/about/guidelines.xhtml

The low specificity after validation of the CR, is probably due to the differences between both datasets (part I and II of the study). The ratio between males and females is different between the two datasets, also the presence of specific ATC codes is different. In addition, the medication dosage and the duration of use is not included in the CR, which may vary between the two datasets. Compared to commonly used fall risk tools, the CR developed in this study, has a higher specificity than the HFRM I tool (sensitivity 97%, specificity 9%) and a higher sensitivity than the STRATIFY tool (sensitivity 63%, specificity 71%) [21]. However, the CR has a lower specificity compared to the STRATIFY tool, and a lower sensitivity compared to HFRM I [21]. When compared to HFRM II (sensitivity 92%, specificity 37%), both sensitivity and specificity are lower [21]. It is important to mention, however, that sensitivity and specificity of the STRATIFY, HFRM I and HFRM II tools are evaluated in older patients in acute care hospitals. Nunan et al. [22] conducted a systematic review to provide a comparison of studies and the results and feasibility of fall risk tools in the long-term care setting. The sensitivity and specificity scores ranged from 50% to 91%, and from 32% to 90% respectively, for eight different multifactorial assessment tools (MATs). One of these MATs was the STRATIFY tool. In the long-term care setting, the STRATIFY tool showed a sensitivity of 50% (vs. 63% in hospital setting) and a specificity of 76% (vs. 71% in hospital setting) [22]. The HFRM I and II tools were not included in the study. The MAT tool with the highest sensitivity in the long-term care setting, was the Downton Index (sensitivity 91%, specificity 39%). The highest specificity was achieved with the Peninsula Health Falls Risk Assessment Tool (PHFRAT) (specificity 90%, sensitivity 58%) [22], however, this tool was validated retrospectively. The CR developed in our study has lower sensitivity and specificity scores compared to both the Downton Index and the PHFRAT.

The rather low AUROC for the prediction model in our study, means that the model is unsuccessful at distinguishing between classes (here: fallers and non-fallers). The optimal AUROC value would be 1.0. In the current study, we found an AUROC of 0.603 (95%CI 0.565-0.641). The low AUROC is probably due to the fact that we choose to base the CR on electronically available data only, in order to make it more suitable for implementation in daily practice. Making this choice, also means that it is not possible to implement important risk factors, like frailty, in the CR. The low AUROC therefore confirms the complex and multifactorial aspects of the risk of fall incidents, since only a few (i.e. gender, age and medication related data) of the fall risk factors were included in the predictive CR.

For peer review only

Besides the included fall risk factors in the developed predictive CR, the intention was to include laboratory data as well, in order to get the most out of the electronically available data. Given the fact that laboratory checks are not performed on daily basis in the nursing home setting, we choose to collect laboratory data up to three months prior to the date of the fall incident. We intended to consider only the most recent known laboratory value of the specific laboratory parameter in the analysis. However, only in approximately 50% of the NH-residents was laboratory data available, for at least one of the laboratory parameters intended for analysis. Taking these data into account in the analysis, would result in a substantial loss of power. Therefore, no association of laboratory data with fall risk was studied.

Regarding the medication data, the presence of specific ATC-codes in the NH-residents medication list was checked. This was carried out at three specific moments: at the day of the fall incident, three days prior to the fall incident and five days prior to the fall incident. Since the association between the number of medications, types of medication, and the risk of falls was studied, medication in use close to the time of the fall incident was collected. It is difficult to define what the exact time period should be, because the effect of a specific medicine on a fall is possibly related to the half-life of the drug; However, it could also result from drug-drug interactions. The half-life of the drug and the presence or absence of drug-drug interactions is different for every resident, therefore, we chose to compare the association at the time of the fall incident and three and five days prior to the fall incident.

Regarding the results on the association of specific ATC codes with fall risk, we only found NO6D (anti-dementia drugs), N06A (antidepressants), N03A (antiepileptics), N05A (antipsychotics), N05C (hypnotics and sedatives), G04 (urologicals), N02A (opioids) and N05B (anxiolytics) to be significantly associated. The other ATC codes were not significant despite their found association in other studies [6,8-20]. However, this could also be due to the fact that some of the ATC codes were not present at all or only for a small group of NH-residents. Furthermore, in our study, polypharmacy was not statistically significantly related to fall risk. Although there are studies that show that polypharmacy increases the risk of falls, a meta-analysis showed that from the 19 studies that investigated the association of polypharmacy in older people with the risk of falls, only 6 studies reported that polypharmacy increases the risk of falling also the odds ratio reporting this association was nearing 1.0 [31]. The question therefore arises whether polypharmacy itself, or only the number of fall risk increasing drugs is a fall risk increasing variable. Further research is needed to investigate this.

For peer review only

For peer review only - http://bmjopen.bmj.com/site/about/guidelines.xhtml

The study has a few limitations. First of all no prospective design was used. In our study we want to know whether people who fall at a certain reference date, have different values for the analyzed variables in the days before the fall (fall incident = reference date) compared to people who do not fall. Since no fall incident date exists for the residents from the control group, we used a pragmatic approach where the data of the residents in the control group were collected at similar dates (i.e. reference dates for controls) as those for the cases. Therefore, each non-faller was linked at random with a faller. However, it should be noted that this reference date could have been any given date for the NH-residents from the control group. In addition, the model (CR) was based on a fall incidence of 50% per year. If this incidence is incorrect, the predicted probabilities of this model are not correct either and we could only assess the C-statistic (AUROC). The conclusion that this model is not clinically useful still holds, as it was based on this C-statistic. Another limitation of the study is that the dataset of the included NH-residents consisted of fallers and non-fallers. The fallers were classified as such based on fall reports. It is unknown whether these fall reports are complete and which definition of a fall incident is used for reporting. It is also known that fall reports which are not fully completed, are not reported. In other words, the number of actual fallers is probably underestimated. Also, for practical reasons we did not collect information on resident characteristics such as mobility device use or the type of disability, etc.

Furthermore, we have chosen not to take into account the history of fall incidents. A risk model for the prevention of recurrent falls in community-dwelling elderly of Stalenhoef et al., showed that two or more falls in the previous year is one of the main determinants of recurrent falls [2]. However, in real life, data about the history of fall incidents is not always available and the reliability of the outcome of the data would be questionable given the information bias in fall incident reports in NH-residents.

The results of our study show that a CR based only on electronically available data is insufficient to predict the fall risk in NH-residents. However, the developed predictive CR could serve as a basis for future research, since more data are made available electronically and this could mean that in the near future more risk factors can be added to the prediction model in order to optimize the model. We would recommend for future research to include medication dosage and duration of medication use in the prediction model as well as frailty and history of falls. Finally, the next step would be to perform a prospectively designed study for validation of the predictive CR in real life NH-residents.

For peer review only

For peer review only - http://bmjopen.bmj.com/site/about/guidelines.xhtml

AUTHOR CONTRIBUTIONS

All authors have made substantial contributions to conception and design, acquisition of data, analysis and interpretation of data; They all have been involved in drafting the manuscript and revising it critically for important intellectual content; They all have given final approval of the version to be published; and they all agree to be accountable for all aspects of the work in ensuring that questions related to the accuracy or integrity of any part of the work are appropriately investigated and resolved.

Conception or design of the work

V Milosevic

AEMJH Linkens

KPGM Hurkens

DR Wong

B Winkens

BPC van Oijen

C Mestres-Gonzalvo

PHM van der Kuy

Data Collection

V Milosevic

PHM van der Kuy

Data analysis and interpretation

V Milosevic

C Mestres-Gonzalvo

BPC van Oijen

B Winkens

PHM van der Kuy

Drafting the article

V Milosevic

For peer review only

For peer review only - http://bmjopen.bmj.com/site/about/guidelines.xhtml

Critical revision of the article

AEMJH Linkens

KPGM Hurkens

B Winkens

BPC van Oijen

C Mestres-Gonzalvo

PHM van der Kuy

Final approval of the version to be published

V Milosevic

AEMJH Linkens

KPGM Hurkens

DR Wong

B Winkens

BPC van Oijen

C Mestres-Gonzalvo

PHM van der Kuy

For peer review only

ETHICAL CONSIDERATIONS (45)

The study was conducted retrospectively using existing data on resident characteristics, laboratory data, medication and reports on fall incidents from nursing home and/or hospital records. The study is approved (METCZ20180135) by the Medical Research Ethics Committee of Zuyderland Medical Centre and Zuyd University of Applied Sciences.

A patient consent form

As the data from the residents was anonymized after extraction no patient consent form was necessary according to the Medical Research Ethics Committee

A data sharing statement

The data underlying this article will be shared on reasonable request to the corresponding author.

For peer review only

For peer review only - http://bmjopen.bmj.com/site/about/guidelines.xhtml

References

[1] WHO Global report on falls prevention in older age, WHO ageing and life course, family and community health, World Health Organization 2007.

[2] Stalenhoef PA, Diederiks JP, Knottnerus JA, *et al*. A risk model for the prediction of recurrent falls in community-dwelling elderly: a prospective cohort study. *J Clin Epidemiol*. 2002 Nov;55(11):1088-94.

[3] Tinetti ME, Speechley M, Ginter SF. Risk factors for falls among elderly persons living in the community. *New England Journal of Medicine*. 1988;319:1701-1707.

[4] Downton JH, Andrews K, Prevalence, characteristics and factors associated with falls among the elderly living at home. *Aging*. 1991;3(3):219-28.

[5] Tinetti ME. Factors associated with serious injury during falls by ambulatory nursing home residents. *Journal of the American Geriatrics Society*. 1987;35:644-648.

[6] Nederlandse Vereniging voor Klinische Geriatrie (NVKG) Richtlijn Preventie van valincidenten bij ouderen (Guideline prevention of fall incidents in elderly patients, from the Dutch association of geriatrics), 2017. Available from: https://www.richtlijnendatabase.nl/richtlijn/preventie_van_valincidenten_bij_ouderen/startpagina_-_preventie_van_valincidenten.html, Feb 2018

[7] Helgadottir B, Laflamme L, Monarrez-Espino J, *et al*. Medication and fall injury in the elderly population: do individual demographics, health status and lifestyle matter? *BMC Geriatric* 2014;14:92

[8] Ham AC, Swart KM, Enneman AW., *et al*. Medication-related fall incidents in an older, ambulant population: I B-PROOF study. *Drugs Aging* 2014;31(12):917-27.

[9] Leipzig RM, Cumming RG, Tinetti ME. Drugs and falls in older people: a systematic review and meta-analysis: I. Psychotropic drugs. *J. Am. Geriatr. Soc.* 1999;47(1):30-9

[10] Leipzig RM, Cumming RG, Tinetti ME. Drugs and falls in older people: a systematic review and meta-analysis: II. Cardiac and analgesic drugs. *J. Am. Geriatr. Soc.* 1999; 47(1):40-50

[11] Woolcott JC, Richardson KJ, Wiens MO, *et al.* Meta-analysis of the impact of 9 medication classes on falls in elderly persons. *Arch Intern Med*. 2009;23;169(21):1952-60

[12] Janus SIM, Reinders GH, van Manen JG, *et al.* Psychotropic drug-related fall incidents in nursing home residents living in the eastern part of the Netherlands. *Drugs R D* (2017) 17:321-328

For peer review only

For peer review only - http://bmjopen.bmj.com/site/about/guidelines.xhtml

[13] Seppala LJ, Wermelink AMAT, de Vries M, *et al*. Fall-risk-increasing drugs: a systematic review and meta-analysis: II. Psychotropics. *J Am Med Dir Assoc.* 2018 Apr;19(4):371.e11-371.e17.

[14] Seppala LJ, van de Glind EMM, Daams JG, *et al.* Fall-risk-increasing drugs: a systematic review and meta-analysis: III. Others. *J Am Med Dir Assoc.* 2018 Apr;19(4):372.e1-372.e8.

[15] de Vries M, Seppala LJ, Daams JG, *et al.* Fall-risk-increasing drugs: a systematic review and meta-analysis: I. Cardiovascular drugs. *J Am Med Dir Assoc.* 2018 Apr;19(4):371.e1-371.e9.

[16] Bor A, Matuz M, Csatordai M, *et al.* Medication use and risk of falls among nursing home residents: a retrospective cohort study. *Int J Clin Pharm* (2017) 39:408-415

[17] Marcum ZA, Perera S, Thorpe JM, *et al.* Antidepressant use and recurrent falls in community-dwelling older adults: Findings from the health ABC study. *Ann Pharmacother.* 2016 July; 50(7):525-533

[18] Berry SD, Placide SG, Mostofsky E, *et al.* Antispychotic and benzodiazepine drug changes affect acute falls risk differently in the nursing home. *Journals of Gerontology: Med sciences,* 2016, Vol. 71, No. 2.

[19] Kuschel BM, Laflamme L, Möller J, The risk of fall injury in relation to commonly prescribed medications among older people-a Swedisch case-control study. *European Journal of Public Health*, Vol. 25, No. 3, 527-532.

[20] Maximos M, Chang F, Patel T, Risk of falls associated with antiepileptic drug use in ambulatory elderly populations: a systematic review. *Can Pharm J* (Ott) 2017; 150:101-111

[21] Matarese M, Ivziku D, Bartolozzi F, *et al.* Systematic review of fall risk screening tools for older patients in acute hospitals. *J Adv Nurs.* 2015 Jun; 71(6):1198-209

[22] Nunan S, Brown WC, Henwood T, *et al.* Fall risk assessment tools for use among older adults in long-term care settings: A systematic review of the literature. *Australas J Ageing.* 2018 Mar;37(1):23-33.

[23] Yazdani C, Hall S, Evaluation of the Medication fall risk score. *Am J Health-Syst Pharm.* 2017; 74:e32-9.

[24] Maurer MS, Burcham J, Cheng H, Diabetes Mellitus Is Associated With an Increased Risk of Falls in Elderly Residents of a Long-Term Care Facility. *Journal of Gerontology: MEDICAL SCIENCES*, 2005, Vol. 60A, No. 9, 1157–1162.

[25] Duh MS, Mody SH, Lefebvre P, *et al.*, Anaemia and the Risk of Injurious Falls in a Community-Dwelling Elderly Population. *Drugs Aging* 2008; 25 (4): 325-334

For peer review only

For peer review only - http://bmjopen.bmj.com/site/about/guidelines.xhtml

[26] Ambrosea AF, Geet P, Hausdorff JM, Risk factors for falls among older adults: A review of the literature. *Maturitas* 75 (2013) 51– 61.

[27] Dharmarajan TS, Avula S, Norkus EP, Anemia Increases Risk for Falls in Hospitalized Older Adults: An Evaluation of Falls in 362 Hospitalized, Ambulatory, Long-Term Care, and Community Patients. *Jamda* 2006.12.015

[28] Polinder-Bos HA, Emmelot-Vonk MH, Gansevoort RT, *et al.* High fall incidence and fracture rate in elderly dialysis patients. *The Netherlands Journal of Medicine*. Dec. 2014, Vol.72, No. 10

[29] Fehlberg EA, Lucero RJ, Weaver MT, Associations between hyponatraemia, volume depletion and the risk of falls in US hospitalized patients: a case–control study. *BMJ Open* 2017;7

[30] Jill Jin, Prevention of Falls in Older Adults, *JAMA*. 2018;319(16):1734

[31] Park H, Satoh H, Miki A, *et al.* Medications associated with falls in older people: systematic review of publications from a recent 5-year period. *Eur J Clin Pharmacol*. 2015 Dec;71(12):1429-40.

For peer review only

Figure 1 caption:

Figure 1: Inclusion of nursing home residents Zuyderland Medical Centre in part I of the study

* Model I is at the reference date (= day of the fall incident), model II is 3 days prior to the reference date and model III is 5 days prior to the reference date.

Figure 2 caption:

Figure 2: ROC curve fall risk predictive clinical rule

For peer review only

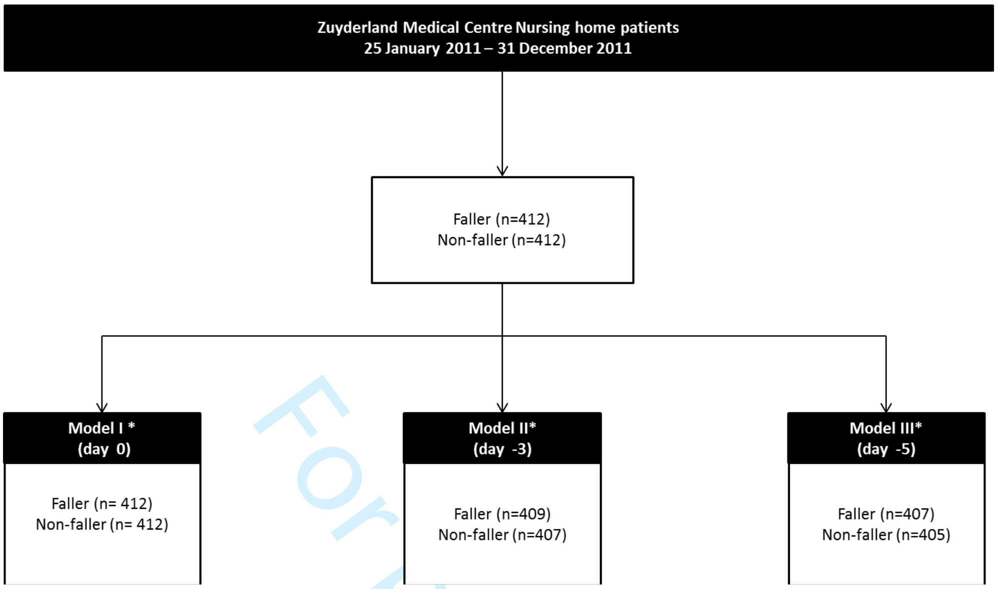

For peer review only

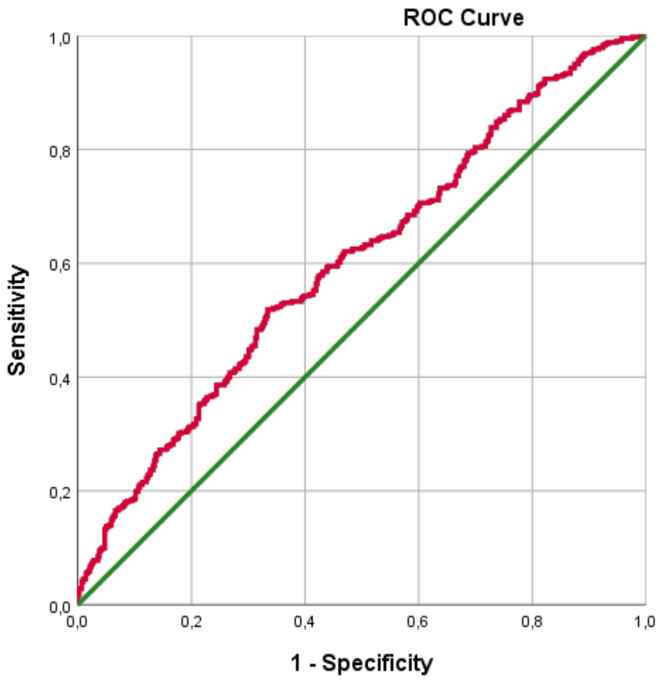

review only

For peer review only - http://bmjopen.bmj.com/site/about/guidelines.xhtml

Supplementary Table 1a: Potential fall risk factors, Model I (day 0)

Risk factors	Mean (SD)	Frequency (number)	% of total	OR	95% C.I. OR		p-value
					Upper	Lower	
**Age (y)**	82.43 (8.46)	-	-	1.044	1.026	1.063	**.000** *
**Age(y) centred**	0.03 (8.46)	-	-	1.035	1.015	1.055	**.001** *
**Age(y) centred^2**	71.57 (147.93)	-	-	.998	.996	.999	**.008** *
**Gender**	-	252 *M*, 572 *F*	30.58 *M*, 69.42 *F*	.561	.415	.758	**.000** *
**ATC unique**	8.49 (3.91)	-	-	1.035	.999	1.072	.059
**ATC unique≥5**		697	84.59	1.183	.810	1.729	.385
**ATC unique 0-5**		127	15.41	.845	.578	1.235	.385
**ATC unique 5- 10**		399	48.42	.864	.657	1.136	.296
**ATC unique 10-15**		226	27.43	1.103	.811	1.499	.531
**ATC unique ≥15**		72	8.74	1.640	1.000	2.690	**.050** *
**N06D**		97	11.77	6.057	3.474	10.558	**.000** *
**C01**		160	19.42	1.168	.827	1.651	.379
**N04A**		1	.12	*NA*	*NA*	*NA*	*NA*
**N06A**		253	30.70	1.394	1.035	1.878	**.029** *
**N03A**		75	9.10	.499	.304	.820	**.006** *
**R06A**		32	3.88	1.139	.561	2.313	.719
**N05A**		304	36.89	2.144	1.605	2.863	**.000** *
**C07**		199	24.15	.864	.628	1.190	.370
**S01ED**		24	2.91	1.414	0.621	3.222	0.409
**N05C**		280	33.98	1.417	1.060	1.894	**.019** *
**G04**		104	12.62	1.635	1.074	2.489	**.022** *
**C08**		80	9.71	.895	.564	1.420	.638
**C03**		281	34.10	1.177	.881	1.571	.270
**M03**		7	.85	.000	.000	*NA*	0.999
**C04AC**		0	.00	*NA*	*NA*	*NA*	*NA*
**C01C**		2	.24	1.000	.062	16.055	1.000
**N02A**		101	12.26	.589	.385	.903	**.015** *
**A02B**		318	38.59	.866	.654	1.147	.316
**C09**		211	25.61	1.039	.760	1.421	.811
**N07C**		20	2.43	1.000	.411	2.435	1.000
**N02B**		395	47.94	1.030	.783	1.355	.834
**N07AA**		3	.36	.498	.045	5.524	.571
**A10**		146	17.72	1.106	.772	1.582	.583
**C02**		6	.73	1.000	.200	4.988	1.000
**M01A**		54	6.55	1.083	.623	1.882	.778
**N07C**		20	2.43	1.000	.411	2.435	1.000
**N05B**		231	28.03	1.352	.996	1.835	**.053** *
**C10**		176	21.36	1.060	.759	1.479	.734
**R01BA**		0	.00	*NA*	*NA*	*NA*	*NA*
**C01A**		69	8.37	1.100	.671	1.804	.705
**C01B**		8	.97	1.000	.248	4.030	1.000
**C01D**		98	11.89	1.149	.753	1.754	.519
**N04**		37	4.49	.945	.488	1.828	.866
**N06DA**		41	4.98	5.217	2.284	11.917	**.000** *
**G04CA**		75	9.10	1.768	1.083	2.885	**.023** *

* statistically significant (p-value < 0.05)

NA = not available; SD = standard deviation; OR = Odds Ratio; C.I.= confidence interval; *M* = male, *F* = female

For peer review only

Supplementary Table 1b: Potential fall risk factors, Model II (day -3)

Risk factors	Mean (SD)	Frequency (number)	% of total	OR	95% C.I. OR		p-value
					Upper	Lower	
**Age (y)**	82.47 (8.39)	-	-	1.043	1.025	1.062	**.000 ***
**Age(y) centred**	0.00 (8.39)	-	-	1.033	1.014	1.054	**.001 ***
**Age(y) centred^2**	70.35 (143.45)	-	-	.998	.996	.999	**.007 ***
**Gender**	-	246 *M*, 570 *F*	30.15% *M*, 69.85% *F*	.546	.403	.740	**.000 ***
**ATC unique**	8.46 (3.90)	-	-	1.029	.993	1.066	.115
**ATC unique≥5**		690	84.56	1.044	.714	1.527	.824
**ATC unique 0-5**		126	15.44	.958	.655	1.401	.824
**ATC unique 5- 10**		401	49.14	.872	.662	1.147	.328
**ATC unique 10-15**		221	27.08	1.057	.776	1.439	.726
**ATC unique ≥15**		68	8.33	1.467	.887	2.428	.136
**N06D**		97	11.89	6.553	3.707	11.584	**.000 ***
**C01**		161	19.73	1.216	.861	1.718	.268
**N04A**		1	.12	*NA*	*NA*	*NA*	*NA*
**N06A**		253	31.00	1.386	1.028	1.868	**.032 ***
**N03A**		75	9.19	.529	.323	.866	**.011 ***
**R06A**		32	3.92	1.133	.558	2.301	.729
**N05A**		304	37.25	2.044	1.530	2.730	**.000 ***
**C07**		199	24.39	.858	.623	1.182	.350
**S01ED**		24	2.94	1.408	.618	3.208	.416
**N05C**		277	33.95	1.365	1.020	1.828	**.036 ***
**G04**		104	12.75	1.554	1.022	2.362	**.039 ***
**C08**		81	9.93	.916	.579	1.450	.708
**C03**		278	34.07	1.132	.847	1.512	.404
**M03**		7	.86	.000	.000	*NA*	.999
**C04AC**		0	.00	*NA*	*NA*	*NA*	*NA*
**C01C**		2	.25	.994	.062	15.954	.996
**N02A**		95	11.64	.568	.366	.883	**.012 ***
**A02B**		314	38.48	.840	.634	1.115	.228
**C09**		211	25.86	1.059	.774	1.449	.720
**N07C**		19	2.33	1.107	.444	2.764	.827
**N02B**		384	47.06	.933	.709	1.229	.624
**N07AA**		3	.37	.495	.045	5.489	.567
**A10**		143	17.52	1.119	.779	1.608	.541
**C02**		6	.74	.994	.199	4.958	.994
**M01A**		53	6.50	.955	.547	1.667	.871
**N07C**		19	2.33	1.107	.444	2.764	.827
**N05B**		229	28.06	1.380	1.015	1.877	**.040 ***
**C10**		179	21.94	1.037	.745	1.446	.828
**R01BA**		0	.00	*NA*	*NA*	*NA*	*NA*
**C01A**		68	8.33	1.133	.688	1.865	.625
**C01B**		8	.98	.994	.247	4.006	.993
**C01D**		100	12.25	1.194	.784	1.816	.409
**N04**		37	4.53	.940	.486	1.818	.853
**N06DA**		40	4.90	6.071	2.519	14.628	**.000 ***
**G04CA**		75	9.19	1.653	1.015	2.689	**.043 ***

* statistically significant (p-value < 0.05)
NA = not available; SD = standard deviation; OR = Odds Ratio; C.I.= confidence interval; *M* = male, *F* = female

For peer review only

Supplementary Table 1c: Potential fall risk factors, Model III (day -5)

Risk factors	Mean (SD)	Frequency (number)	% of total	OR	95% C.I. OR		p-value
					Upper	Lower	
**Age (y)**	82.52 (8.36)	-	-	1.042	1.024	1.061	**.000** *
**Age(y) centred**	0.00 (8.36)	-	-	1.033	1.013	1.053	**.001** *
**Age(y) centred²**	69.75 (143.05)	-	-	.998	.996	.999	**.010** *
**Gender**	-	243 *M*, 569 *F*	29.93 *M*, 70.07 *F*	.536	.395	.728	**.000** *
**ATC unique**	8.44 (3.93)	-	-	1.023	.987	1.059	.214
**ATC unique≥5**		684	84.24	1.083	.742	1.581	.679
**ATC unique 0-5**		128	15.76	.923	.633	1.348	.679
**ATC unique 5- 10**		395	48.65	.906	.688	1.194	.484
**ATC unique 10-15**		223	27.46	1.083	.796	1.474	.613
**ATC unique ≥15**		66	8.13	1.296	.781	2.152	.316
**N06D**		94	11.58	6.833	3.801	12.285	**.000** *
**C01**		160	19.70	1.198	.847	1.695	.306
**N04A**		1	.12	*NA*	*NA*	*NA*	*NA*
**N06A**		251	30.91	1.357	1.006	1.830	**.045** *
**N03A**		74	9.11	.541	.330	.888	**.015** *
**R06A**		30	3.69	.995	.480	2.063	.989
**N05A**		298	36.70	1.973	1.475	2.639	**.000** *
**C07**		196	24.14	.868	.629	1.198	.391
**S01ED**		24	2.96	1.408	.618	3.208	.416
**N05C**		273	33.62	1.400	1.045	1.878	**.024** *
**G04**		103	12.68	1.457	.958	2.215	.078
**C08**		78	9.61	.940	.589	1.499	.794
**C03**		277	34.11	1.120	.837	1.498	.446
**M03**		7	.86	.000	.000	*NA*	.999
**C04AC**		0	.00	*NA*	*NA*	*NA*	*NA*
**C01C**		2	.25	.994	.062	15.955	.996
**N02A**		91	11.21	.586	.374	.916	**.019** *
**A02B**		312	38.42	.840	.633	1.114	.226
**C09**		209	25.74	1.060	.773	1.451	.719
**N07C**		19	2.34	1.107	.444	2.764	.827
**N02B**		383	47.17	.905	.687	1.194	.481
**N07AA**		3	.37	.495	.045	5.489	.567
**A10**		140	17.24	1.065	.739	1.534	.736
**C02**		6	.74	.994	.199	4.959	.994
**M01A**		54	6.65	.918	.528	1.597	.761
**N07C**		19	2.34	1.107	.444	2.764	.827
**N05B**		228	28.08	1.332	.980	1.812	.067
**C10**		175	21.55	1.038	.743	1.451	.826
**R01BA**		0	.00	*NA*	*NA*	*NA*	*NA*
**C01A**		68	8.37	1.133	.688	1.865	.625
**C01B**		8	.99	.994	.247	4.006	.993
**C01D**		99	12.19	1.168	.766	1.781	.469
**N04**		37	4.56	.940	.486	1.818	.853
**N06DA**		39	4.80	7.307	2.827	18.885	**.000** *
**G04CA**		75	9.24	1.555	.958	2.524	.074

* statistically significant (p-value < 0.05)
NA = not available; SD = standard deviation; OR = Odds Ratio; C.I.= confidence interval; *M* = male, *F* = female

For peer review only

For peer review only - http://bmjopen.bmj.com/site/about/guidelines.xhtml

TRIPOD

TRIPOD Checklist: Prediction Model Development

Section/Topic	Item	Checklist Item	Page
**Title and abstract**			
Title	1	Identify the study as developing and/or validating a multivariable prediction model, the target population, and the outcome to be predicted.	1
Abstract	2	Provide a summary of objectives, study design, setting, participants, sample size, predictors, outcome, statistical analysis, results, and conclusions.	3
**Introduction**			
Background and objectives	3a	Explain the medical context (including whether diagnostic or prognostic) and rationale for developing or validating the multivariable prediction model, including references to existing models.	5-6
	3b	Specify the objectives, including whether the study describes the development or validation of the model or both.	6
**Methods**			
Source of data	4a	Describe the study design or source of data (e.g., randomized trial, cohort, or registry data), separately for the development and validation data sets, if applicable.	7
	4b	Specify the key study dates, including start of accrual; end of accrual; and, if applicable, end of follow-up.	7
Participants	5a	Specify key elements of the study setting (e.g., primary care, secondary care, general population) including number and location of centres.	7
	5b	Describe eligibility criteria for participants.	7
	5c	Give details of treatments received, if relevant.	-
Outcome	6a	Clearly define the outcome that is predicted by the prediction model, including how and when assessed.	7
	6b	Report any actions to blind assessment of the outcome to be predicted.	-
Predictors	7a	Clearly define all predictors used in developing or validating the multivariable prediction model, including how and when they were measured.	7-8
	7b	Report any actions to blind assessment of predictors for the outcome and other predictors.	-
Sample size	8	Explain how the study size was arrived at.	8-9
Missing data	9	Describe how missing data were handled (e.g., complete-case analysis, single imputation, multiple imputation) with details of any imputation method.	13-16
Statistical analysis methods	10a	Describe how predictors were handled in the analyses.	7-9
	10b	Specify type of model, all model-building procedures (including any predictor selection), and method for internal validation.	7-9
	10d	Specify all measures used to assess model performance and, if relevant, to compare multiple models.	7-9
Risk groups	11	Provide details on how risk groups were created, if done.	-
**Results**			
Participants	13a	Describe the flow of participants through the study, including the number of participants with and without the outcome and, if applicable, a summary of the follow-up time. A diagram may be helpful.	figure 1
	13b	Describe the characteristics of the participants (basic demographics, clinical features, available predictors), including the number of participants with missing data for predictors and outcome.	table 1
Model development	14a	Specify the number of participants and outcome events in each analysis.	suplementary tables
	14b	If done, report the unadjusted association between each candidate predictor and outcome.	suplementary tables
Model specification	15a	Present the full prediction model to allow predictions for individuals (i.e., all regression coefficients, and model intercept or baseline survival at a given time point).	table 3
	15b	Explain how to the use the prediction model.	table 3
Model performance	16	Report performance measures (with CIs) for the prediction model.	table 4
**Discussion**			
Limitations	18	Discuss any limitations of the study (such as nonrepresentative sample, few events per predictor, missing data).	13-16
Interpretation	19b	Give an overall interpretation of the results, considering objectives, limitations, and results from similar studies, and other relevant evidence.	13-16
Implications	20	Discuss the potential clinical use of the model and implications for future research.	13-16
**Other information**			
Supplementary information	21	Provide information about the availability of supplementary resources, such as study protocol, Web calculator, and data sets.	-
Funding	22	Give the source of funding and the role of the funders for the present study.	4

We recommend using the TRIPOD Checklist in conjunction with the TRIPOD Explanation and Elaboration document.
